# Aliphatic Chains and External Pressure as Tools for Fine-Tuning Spin Transition Temperature and Cooperativity

**DOI:** 10.1021/acs.inorgchem.5c03403

**Published:** 2025-10-09

**Authors:** Hanlin Yu, Maksym Seredyuk, Kateryna Znovjyak, Nikita Liedienov, Wei Xu, Francisco Javier Valverde-Muñoz, M. Carmen Muñoz, Joachim Kusz, Maria Książek, Ruixin Li, Quanjun Li, Bingbing Liu, Gábor Molnár, Georgiy Levchenko, José Antonio Real

**Affiliations:** † State Key Laboratory of High Pressure and Superhard Materials, 12510 Jilin University, 130012 Changchun, China; ‡ Department of Chemistry, Taras Shevchenko National University of Kyiv, 01601 Kyiv, Ukraine; § Donetsk Institute for Physics and Engineering named after O.O. Galkin, NASU, 03028 Kyiv, Ukraine; ∥ State Key Laboratory of Inorganic Synthesis and Preparative Chemistry, College of Chemistry, Jilin University, 130012 Changchun, China; ⊥ CNRS IPR—UMR 6251, Univ Rennes, F-35000 Rennes, France; # Departamento de Fisica Aplicada, 16774 Universitat Politècnica de València, Camino de Vera s/n, 46022 Valencia, Spain; ∇ Institute of Physics, 49568 University of Silesia, 75 Pułku Piechoty 1, 41-500 Chorzów, Poland; ○ School of Physics Science and Information Technology, Liaocheng University, 252000 Liaocheng, China; ◆ 54919 Laboratoire de Chimie de Coordination CNRS & Université de Toulouse (UPS, INP), 205 Route de Narbonne, 31077 Toulouse, France; ¶ International Center of Future Science, Jilin University, 130012 Changchun, China; †† Instituto de Ciencia Molecular, Departamento de Química Inorgánica, Universidad de Valencia, 46980 Paterna, Valencia, Spain

## Abstract

In switchable spin crossover (SCO) molecular materials, control of their critical temperature and cooperativeness is essential for practical applications. This requires systematic fine-tuning of the molecular packing in the crystal. Therefore, here we report on the synthesis and characterization of a series of Fe^II^ SCO complexes, {Fe^II^L}­(ClO_4_)_2_, derived from a tripodal tren-based imino-methyl-pyridine hexadentate-type ligand L with alkoxy chains of varying lengths (**C3** to **C22**). The complexes crystallize in the monoclinic *P*2_1_/c space group, displaying an identical coordination core. The crystal packing, modulated by the length of the alkoxy chains, is governed by the amphiphilic nature of the complexes, where the hydrophilic pseudo-octahedral cationic heads and ClO_4_^–^ anions define double-layers separated by the growing hydrophobic layer generated by the alkoxy chains. All compounds undergo thermal- and photoinduced SCO behavior featuring transition temperatures and cooperativeness which depend, for most of them, on the odd–even parity of the chains. The pressure dependence of the SCO for selected members (**C3**, **C8**, **C15**, and **C16**) has been investigated through magnetic, UV–vis, and IR measurements. Both internal pressure generated by the growth of the aliphatic layer and the applied external pressure effects are compared and discussed in the context of fine-tuning modulation of SCO properties.

## Graphical abstract

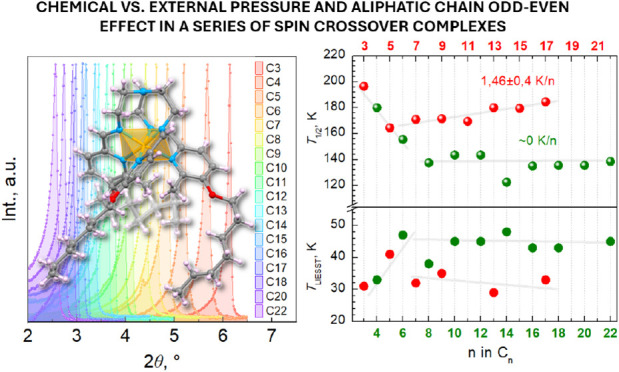

## Introduction

Spin Crossover (SCO) compounds represent a class of switchable materials that exhibit reversible transitions between low-spin (LS) and high-spin (HS) states under external stimuli such as temperature, pressure, or light irradiation.^1^ These bistable systems hold significant potential for applications in memory devices, molecular switches, or in display technologies due to their ability to undergo abrupt and hysteretic transitions near room temperature (RT).^2^ The SCO in Fe^II^ complexes is typically accompanied by distinct structural changes, particularly in [FeN_6_] octahedral coordination environment, where the average Fe–N bond length increases by ≈0.2 Å upon LS-to-HS state switching.^3^ These structural distortions propagate through the crystal lattice via intermolecular interactions (*e.g*., π–π stacking, hydrogen bonding, and van der Waals forces) or/and covalent linkages, influencing the cooperativity of the transition.^4^ In particular, the cooperativity is viewed as essential for achieving sharp transitions with hysteresis, a critical feature for practical applications.^5^

Chemical pressure imposed by crystal packing constraints provides a subtle yet important means of tuning SCO behavior.^6^ This internal pressure stemming from the intermolecular interactions, influences the lattice ability to accommodate structural changes during the transition.^7^ By carefully designing ligand backbones and crystal packing arrangements, the internal pressure can be used to systematically adjust transition temperatures, offering a pathway to tailor SCO characteristics without external stimuli. Complementary to intrinsic structural factors, *external pressure* serves as an efficient tool for manipulating spin states. Hydrostatic pressure can substantially alter SCO, often inducing transitions in compounds that otherwise remain locked in the HS state across the entire temperature range.^8^ This phenomenon arises from pressure-induced reduction of the metal–ligand bonds, effectively increasing the ligand-field strength and stabilizing the LS state. Such effects can lead to significant modifications in transition completeness, almost always shifting critical temperatures, and even transform gradual transitions into abrupt ones or vice versa.^9^ The influence of external and chemical pressure on the LS-HS transformation highlights the delicate balance between ligand-field effects and lattice elasticity in SCO systems.^10^ While the hydrostatic pressure offers a direct and reversible means of perturbing the spin equilibria, chemical pressure provides a built-in mechanism for fine-tuning material properties at the molecular level. Together, these pressure effects expand the toolbox for developing materials with precisely controlled switching SCO behaviors.

In this context, the development of SCO Fe^II^ complexes derived from tren-based tripodal hexadentate ligands offers a compelling platform to explore how chemical modifications influence SCO responses. These systems have proven to be especially useful for investigating how subtle variations in crystal packing, introduced through pendant substituents of varying nature, can dramatically affect SCO behavior, often in nonlinear and cooperative ways. Notably, the design flexibility of these ligands enables the integration of additional solid-state features such as homochirality, redox activity, mixed valence, second-order nonlinear optical responses, and counterion-dependent supramolecular organization.^11^ For example, we have reported systems like {Fe^II^[tren­(6F-py)_3_]}­(X)_2_ (X = BF_4_^–^, ClO_4_^–^), where a phase transition disrupts the inherent gradual SCO, producing a strong asymmetric cooperative effect.^12^ These findings illustrate how chemical pressure, through rational ligand design and controlled functionalization, can be harnessed not only to modulate SCO but also to link it with other desirable material properties. Moreover, the amenability of the tripodal ligands to chemical modification by aliphatic chains has enabled studies into crystal-to-liquid crystal phase transitions and their reciprocal influence on SCO behavior,^13^ thereby bridging molecular-level tunability with macroscopic functionality.

Predicting how ligand structure variation impacts packing and SCO parameters is important for designing SCO-based functional materials. While using aliphatic chains to modify crystal structures in SCO compounds is well-known, systematic studies where only chain length varies are limited. Short aliphatic chains can significantly alter lattice arrangements and produce either monotonous^14^ or nonmonotonous change in SCO behavior,^15^ as shown by single crystal and magnetic data of different types of substituted ligands. In contrast, long aliphatic chains typically create defined aliphatic sublattices, with chain length adjustments having subtler structural effects, but still able to tune transition temperature, completeness,^16^ or even induce multistep^15a,17^ or reverse^18^ SCO transitions.

In this work, we focus on a family of SCO Fe^II^ *tren*-complexes with three 5-alkoxy-6-methyl-pyridyl pendants, which, opposite to the reported up to now series of compounds, share the same crystal structure and packing arrangement but differ in aliphatic chain length, ranging from short propyl- to long docosanyl-oxy substituents (Scheme 1). This design enables investigation of the effect of chain elongation while keeping other structural variables constant. We report their synthesis, structural characterization, spectral and magnetic properties, and, in addition, we examine the effect of external hydrostatic pressure on their SCO behavior, to get a better understanding of how subtle structural modifications and external stimuli collectively influence the switching properties of these compounds.

**Scheme 1:**
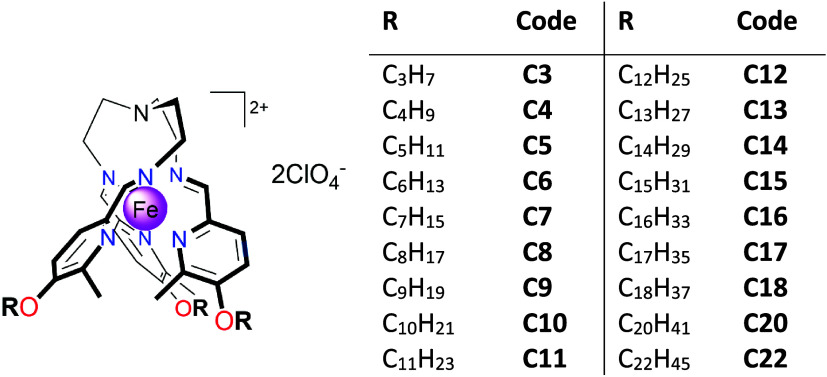
Schematic Structure and Substituents of the Title Compounds

## Results and Discussion

### Magnetism and Mössbauer Spectroscopy

At RT, compounds **C3**–**C22** are HS, but the χ_M_ *T* product decreases down upon cooling due to the SCO process and reaches values close to zero at low temperature. The only exceptions are long chain compounds **C18**, **C20** and **C22**, whose χ_M_ *T* value does not reach the zero level thereby indicating an incomplete transition with up to one-third of molecules remaining in the HS state (Figure S1). Upon heating, the χ_M_ *T* product follows the curve progression obtained in the cooling mode except for **C7**, which displays a hysteresis of the SCO with the loop 10 K wide. Also, **C8** displays an abrupt transition without hysteresis. The characteristic *T*_1/2_ value, at which the molar fractions of the HS and LS molecules are equal to 50%, is clearly dependent on the length and the parity of the carbon atoms of the aliphatic chains (Figure 1). The *T*_1/2_ values plotted against the number of carbon atom *n* of the aliphatic chains can be divided into three subgroups. For the first subgroup with the short chains (**C3**–**C6**), the *T*_1/2_ progressively drops from 188 K down to 150 K. With further increasing n-values, the odd- and even-numbered chains values bifurcate. The even-numbered subgroup formed by **C8**–**C22**, exhibits almost a constant value of *T*_1/2_ ≈ 138 K, while, in contrast, for the odd-numbered chain compounds **C7**–**C17**, a trend to increasing *T*_1/2_ value is evident in the range 167–185 K with the average rate 1.46 ± 0.4 K/n. The difference in *T*_1/2_ between the neighbor odd- and even-numbered chains varies in the range 20–40 K.

**Figure 1:**
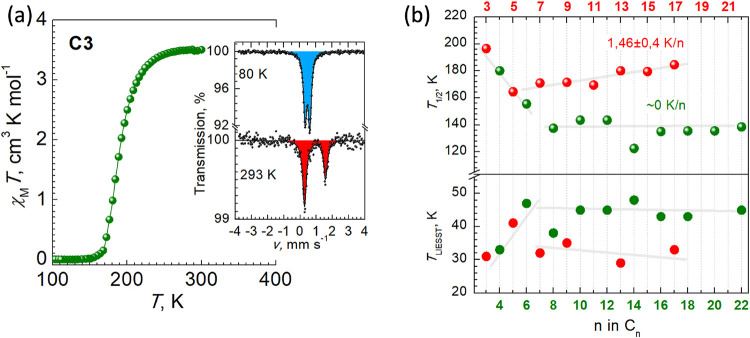
(a) Plot χ_M_ *T* vs *T* for **C3** with an inset of the Mössbauer spectra at the indicated temperature; (b) plot *T*_1/2_ vs *n* and *T*_LIESST_ vs *n* for **C3**–**C22**.

Upon irradiation at 10 K with a red laser (633 nm, 15 mW cm^–1^), **C3**–**C22** compounds exhibit LIESST effect^19^ (Figure S1), characterized by critical relaxation temperatures (*T*_LIESST_) (see Figure 1) that, generally, are dependent on *T*_1/2_ values, thereby reflecting the revealed grouping by chain length/parity and the inverse energy gap rule.^20^ The *T*_LIESST_ increases in the range 31–48 K for **C3**–**C6**, but thereafter the longer-chain compounds diverge by parity. For longer even-chain compounds, the *T*_LIESST_ temperatures are around 45 K, while for odd-chains, being clearly smaller, oscillate in the interval 32–35 K. Also, the long even-chain compounds exhibit almost complete excitation, while for the odd-chains starting from **C9** a relaxation is observed immediately after the laser is turned off.

Mössbauer spectra were intentionally measured for compounds **C3**, **C7** with known single-crystal structures for direct correlation of hyperfine parameters and structural features, and **C12–C14** as the first representatives of longer chain compounds with a consolidated aliphatic sublattice (see below). The Mössbauer spectrum of **C3** at 80 K exhibits a slightly asymmetric resonance signal, deconvolved into a single LS doublet with an isomer shift (δ) of 0.47(1) mm s^–1^ and quadrupole splitting (Δ*E*_Q_) of 0.29(0) mm s^–1^ (Figure 1a, inset). At 293 K, the soft nature of the compound results in a weak resonance signal. Supported by magnetic and structural data, the spectrum was fitted with a single asymmetric doublet, yielding δ = 0.91(1) mm s^–1^ and Δ*E*_Q_ = 1.29(2) mm s^–1^, consistent with the HS state.^11g,13b,13d^ For longer-chain compounds **C7, C12–C14**, the collection of the data was challenging in the HS state, likely due to a low Debye–Waller factor, therefore the measurements were performed only in the LS state at 80 K. Analysis of the results reveals subtle differences in fit parameters based on chain length parity (Table S5 and Figure S2). The Δ*E*_Q_ for odd-numbered chains (**C3**, **C7** and **C13**) is consistently larger by 0.01–0.02 mm s^–1^ than for even-numbered chains (**C12**, **C14**), reflecting variations in the electric field gradient (EFG). In the LS state, the EFG is primarily influenced by lattice contributions from the noncubic environment.^21^ Despite identical coordination head-groups and Fe^II^ spin states, these differences arise from variations in the relative positions of terminal aliphatic substituents and their intermolecular interactions. This effect, consistent across longer-chain compounds (**C9**–**C22**), influences both the quadrupole splitting and the cooperativity parameter discussed below.

### Calorimetry

The thermal dependence of the heat capacity for the representative compounds **C3** and **C7** was monitored through differential scanning calorimetric (DSC) measurements recorded at 10 K min^–1^ (Figure 2a,b, respectively). For **C3**, the average enthalpy Δ*H* and entropy variations Δ*S* (= Δ*H*/*T*_c_) (*T*_c_ is the temperature at the maximum/minimum of Δ*C*_p_ vs *T* plot) associated with the exo- and endothermic peaks (Figure 2a) are, respectively, 15.6 kJ mol^–1^ and 84.2 J K^–1^ mol^–1^. These Δ*H* and Δ*S* values are consistent with the occurrence of a cooperative complete SCO.^22^ The *T*_c_^av^ = 183.5 K calculated as average of the values derived from the maximum/minimun of the Δ*C*_p_ vs *T* curves, in the cooling/heating modes, respectively, is lower than obtained from magnetic measurements (196 K), and reflects the temperature at which most of the heat required by the SCO has come into play in the broad SCO process. For **C7**, the DSC curves reveal two superimposed signals, with maxima/minima on cooling/heating at 166/175 K and 191/191 K (Figure 2b), corresponding to the steep and gradual parts of the magnetic curves, respectively (see Figure S1). The total enthalpy Δ*H* and entropy variations Δ*S* (= Δ*H*/*T*_c_) (*T*_c_ is the average at maxima, 181 K) are, respectively, 17.5 kJ mol^–1^ and 96.7 J K^–1^ mol^–1^.

**Figure 2:**
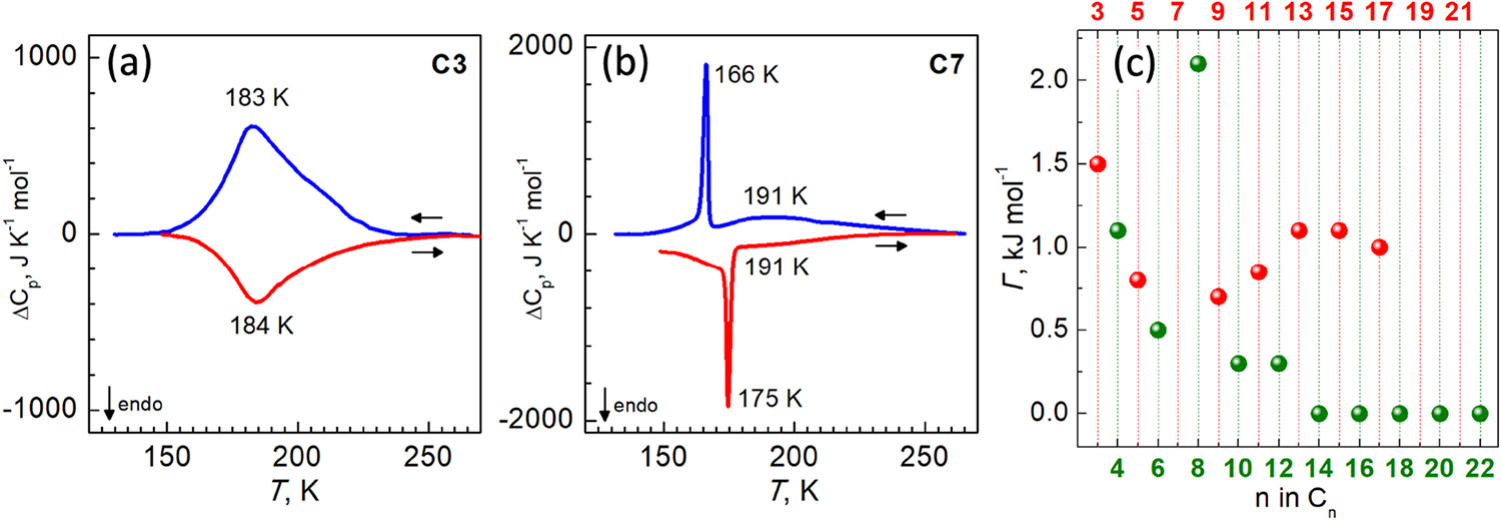
Variation of the heat capacity Δ*C*_p_ associated with the SCO process of **C3** (a) and **C7** (b) in cooling and heating modes. Dependence of the SCO cooperativity Γ vs length of the aliphatic substituents *n* (c).

To quantify the abruptness of the SCO, the magnetic curves of compounds with one-step SCO were simulated using the following equation derived from the regular solution model

$$\left[\ln\left(1-\gamma_{\mathrm{HS}}\right)/\gamma_{\mathrm{HS}}\right]=\left[\Delta H+\Gamma \left(1-2\gamma_{\mathrm{HS}}\right)\right]/\mathrm{RT}-\Delta S/R$$

where γ_HS_ is the HS molar fraction, *R* is the gas constant, Δ*H* and Δ*S* are the experimental enthalpy and entropy change associated with the SCO, respectively, and Γ corresponds to the interaction parameter, accounting for cooperativity.^23^ The Δ*S* values for all compounds were fixed to the experimental value for **C3** to enable a rough assessment of the variation in Γ across the series (Figures 2c and S1). For **C3**, the cooperativity is 1.5 kJ mol^–1^, and it is linearly decreasing for the next members of the series. A jump-like increase in the value is observed for **C8**, whereas beyond this point, the trend diverges according to the parity of the chain length. For longer even-numbered compounds (**C10**–**C22**), the cooperativity decreases steadily and approaches zero by **C14** and remains negligible through **C22**. In contrast, the odd-numbered series (**C9**–**C17**) demonstrates a distinct pattern where cooperativity initially increases, reaches a local maximum, and then declines. This bifurcation also highlights a distinct odd–even effect in the cooperative behavior of the compounds.

### Diffraction and Spectroscopic Data

The complexes crystallize from hot methanol as plate-like red-orange thin crystals (**C3**–**C17**) or as fine pearlescent pale orange precipitates (**C18**–**C22**), whose color intensity decreases with the increase in length of the aliphatic chain. All compounds are strongly thermochromic, turning dark violet upon cooling below the transition temperature and restoring initial lighter coloration upon heating to RT.

Single-crystal X-ray diffraction analyses of **C3** at 100 and 250 K, and of **C7** at 120 and 250 K, reveal that both structures adopt the monoclinic *P*2_1_/*c* centrosymmetric space group (Table S1). For **C3**, the unit cell parameters *a*, *b*, and *c* increase upon heating in the temperature range 100–260 K with relative changes of Δ*a* = +3.0%, Δ*b* = +1.9%, and Δ*c* = +1.3% (Table S2 and Figure S3). Over this range, the unit cell volume (*V*) increases by +6.5%, with an inflection point at 190 K that closely matches the SCO temperature derived from magnetic measurements (196 K). Notably, the monoclinic angle β varies nonlinearly between 89.6° and 91.1°, occasionally attaining the ideal 90° (Figure S3).

Figure 3 shows the molecular structures of **C3** and **C7** at low temperature and of the already known compound **C6** ^13b^ included in the work for comparison. They have an identical coordination core with the Fe^II^ ion octahedrally surrounded by three Fe–N^imine^ and three Fe–N^py^ bonds of the hexadentate Schiff-base ligand (Figure S4). The crystal lattice contains a racemic Δ–Λ mixture of chiral complex cations featuring similar average bond lengths in both spin states (see Table 1). The octahedral distortion parameters ∑, Θ,^24^ continuous shape measures [CShM’s­(O_h_)],^25^ and octahedral volume *V*_O_h__ are more similar between odd-chain **C3** and **C7** than with even-chain **C6** (Table 1). The most pronounced differences are observed in the conformations of terminal segments of aliphatic chains, which tend to adopt an extended (all-*trans*) conformation as the chain length increases. For the neighbors **C6** and **C7** at 120 K, representing even- and odd-chain compounds, the spatial positioning of terminal aliphatic groups and the number of intermolecular interactions, such as weak C–H···C/N/O bonds, differ. These interactions are more numerous for **C6** (Table S4), which is attributed to the parity effect. This difference in intermolecular bonding might be the reason for the distinct magnetic behavior of the compounds. If for **C6** in the HS state the aliphatic chains and anions remain ordered, accompanied just by an increase in thermal ellipsoids, for **C7** under these circumstances, additionally, all aliphatic chains and one anion become disordered (see Figure S5). Coupling of the mechanical stress due to the contraction/expansion of the coordination sphere [FeN_6_] and order–disorder phenomena of the peripheral groups/anions enhances the elastic interactions within the lattice, and can lead to the hysteretic behavior.^12,26^ Apparently, this is the reason for the hysteresis of SCO in **C7** too in contrast to **C6**, for which the absence of such disorder results in gradual transitions without hysteresis.

**Figure 3:**
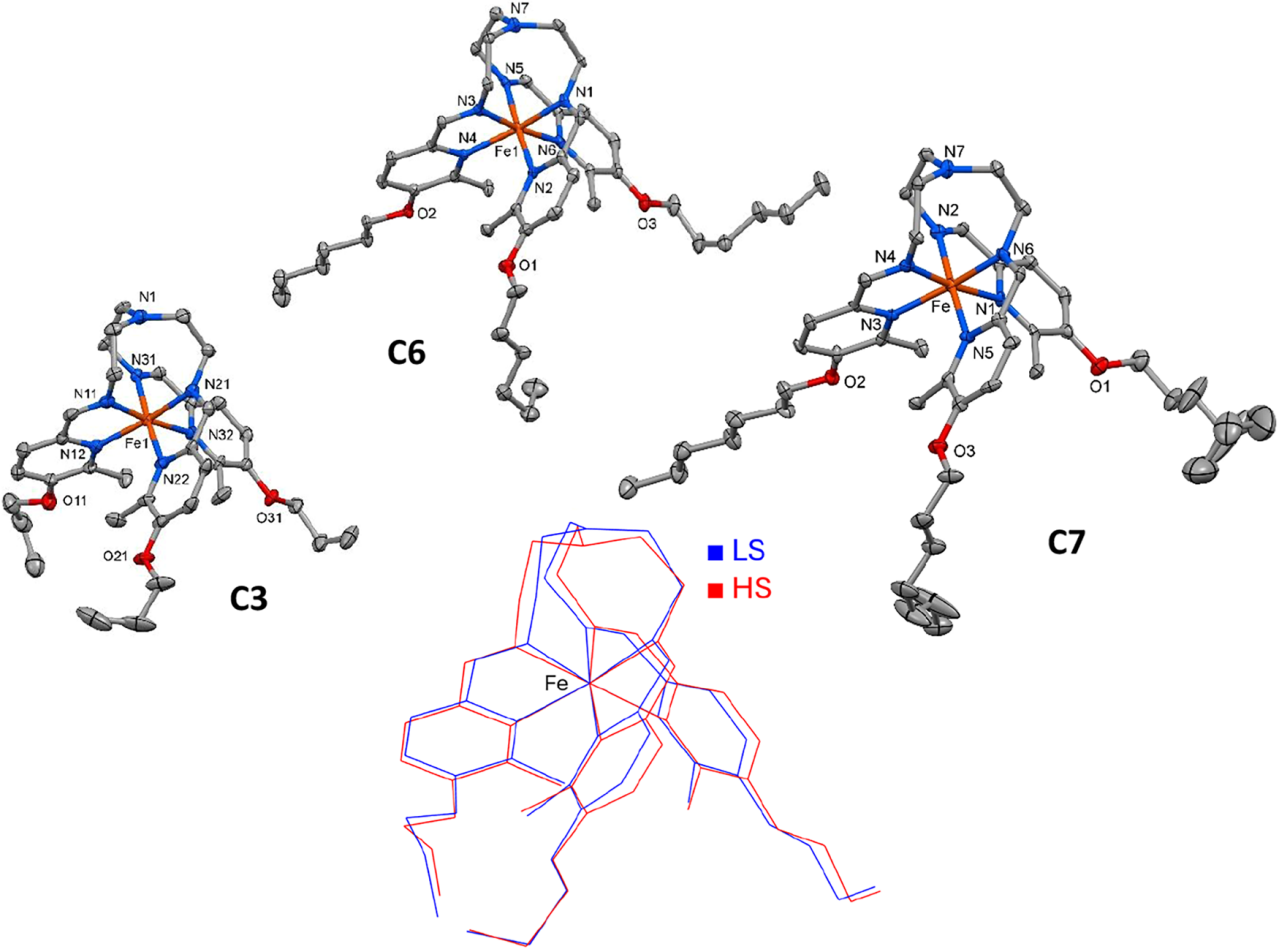
Projection of the indicated complex cations at low temperature in the LS state. Hydrogen atoms and perchlorate anions are omitted for clarity. Displacement ellipsoids are shown at a 30% probability level. At the bottom, the overlay shows the molecular changes of **C3** due to the SCO.

**Table 1:** Distortion Parameters in the LS/HS States of the Indicated Compounds

	*T*, K	spin state	⟨Fe–*N*⟩, Å	*V*(O_h_), Å^3^	∑, deg	Θ, deg	CShM(O_h_)
**C3**	100/250	LS/HS	2.020/2.218	10.653/13.745	86.70/115.84	236.99/344.92	1.227/2.174
**C6** ^a^	90/298	LS/HS	2.007/2.234	10.444/14.039	85.56/116.60	231.29/347.87	1.212/2.246
**C7**	120/250	LS/HS	2.011/2.220	10.503/13.779	86.43/115.10	234.25/341.88	1.223/2.142

a Values calculated from the structural data in ref 13b.

The crystal packing is governed by the amphiphilic nature of the complexes. Consequently, the hydrophilic pseudo-octahedral cationic heads and the tetrahedral anions ClO_4_^–^ are segregated, defining slightly corrugated double-layers that extend parallel to the *bc* plane and stack along *a*. The molecules of one single-layer are oppositely oriented to those of the other single-layer and, consequently, the aliphatic tails, pointing toward opposite directions along *a*, define the hydrophobic intermediated space between two consecutive double-layers (see Figure 4). Within each double layer, the perchlorate anions localize in similar spatial positions for **C3**, **C6**, and **C7**.

**Figure 4:**
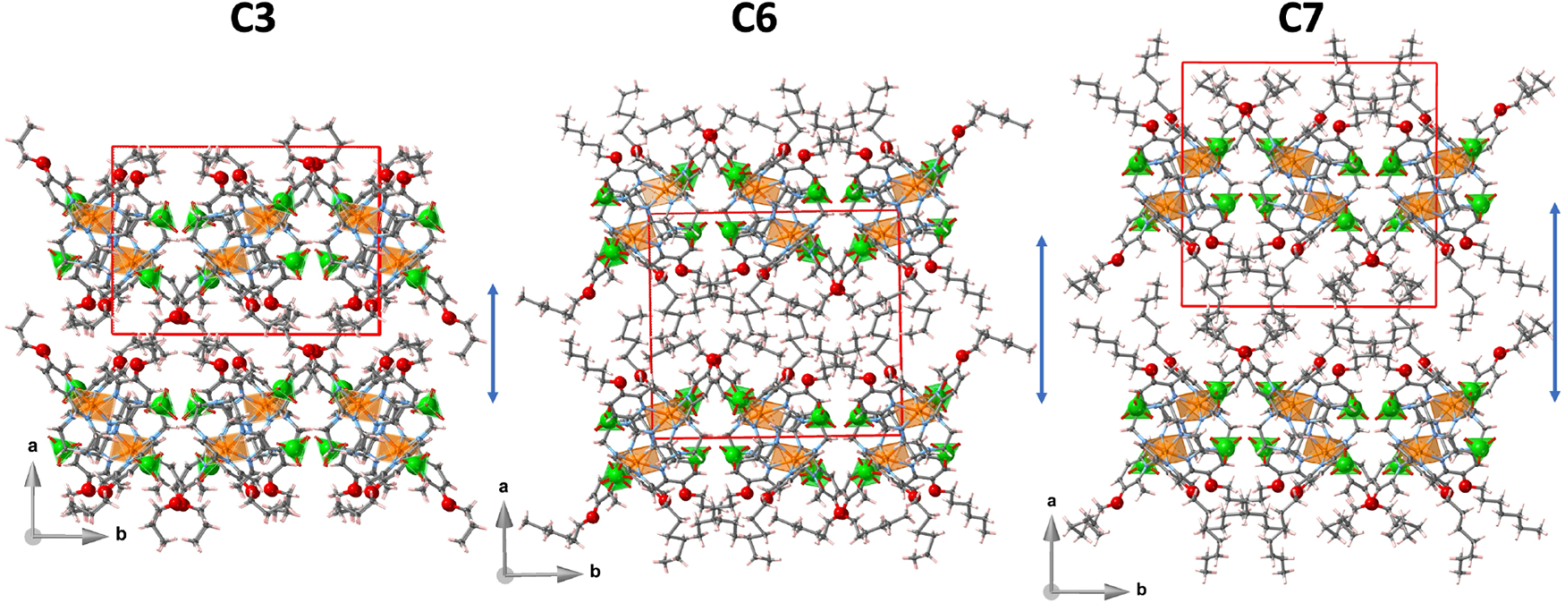
Projection of the packing of the indicated compounds. The double arrow emphasizes the growth of the aliphatic layer.

This supramolecular organization affords the main common feature of the whole series, namely the very intense [100] reflection, commonly observed in other SCO systems containing aliphatic sublattices,^13d,16a,27^ that determines the separation between two consecutive double layers (Figure S6). Obviously, as the number of C atoms increases in the series **C3**–**C22**, the separation between the double-layers, corresponding to the *a* parameter, increases at an average rate of *ca*. 1 Å per carbon atom (see Figure 5). It deserves to be noted that this increase is not monotonous, as the variation of *a* for **C6**–**C18** is systematically slightly larger for odd chains, which is clearly seen on the plot with the subtracted slope (Figure 5).

**Figure 5:**
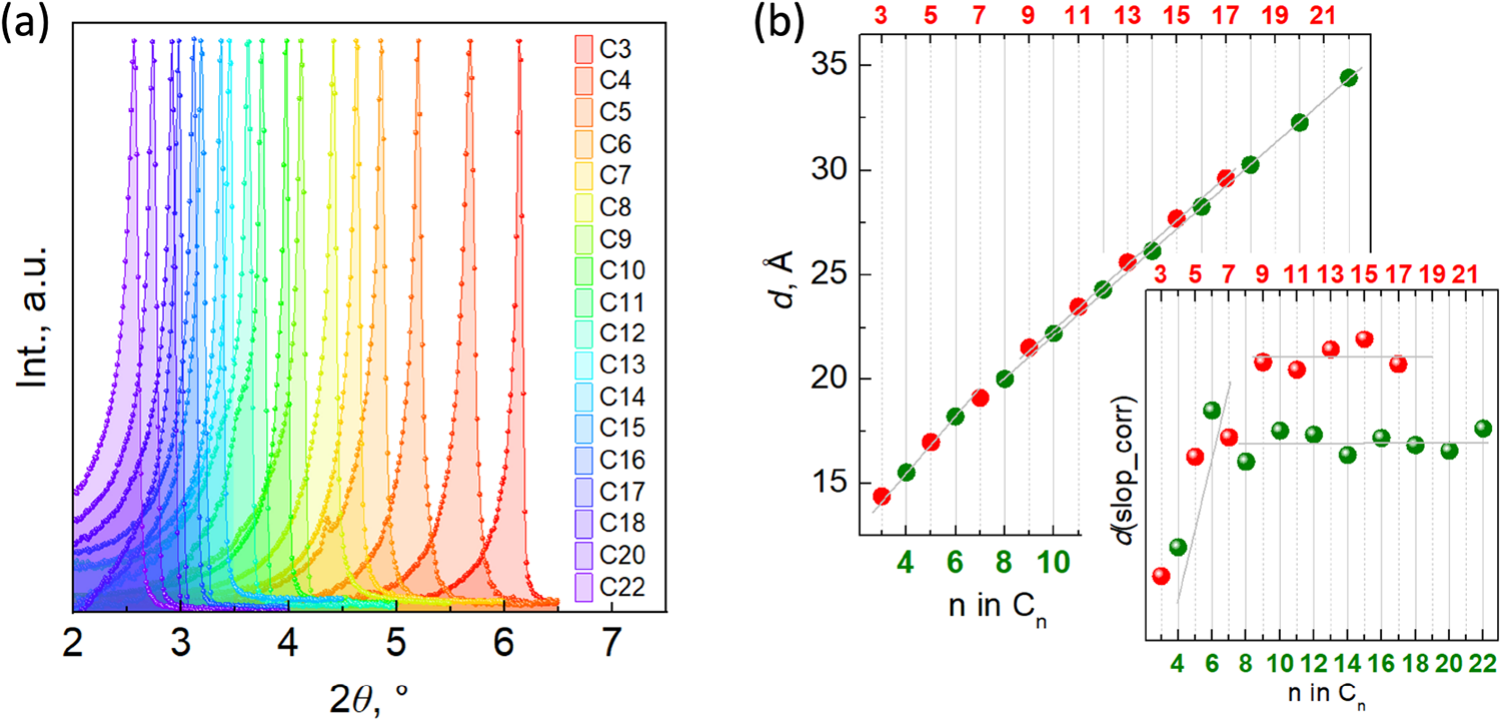
(a) Intense reflection [100] in the series; (b) plot of the unit cell parameter *a* as a function of *n*, with an inset showing the same plot with the slope subtracted.

To gain a deeper understanding of the lattice organization, particularly in longer chain congeners, IR spectra were recorded (Figure S8). The absorption bands in the region characteristic of C–H stretching vibrations indicate increasing conformational order of the aliphatic chains within the lattice as n increases (Figure 6). For **C3**, the asymmetric and symmetric C–H stretching frequencies of methylene groups (ν_asym_(C–H) = 2936 cm^–1^ and ν_sym_(C–H) = 2878 cm^–1^) suggest a *gauche* conformation of methylene groups consistent with the single-crystal structure of the compound. As the chain length increases, the peak maxima systematically shift up, indicating a growing *trans* conformer population, as also observed for the crystal structures of **C6** and **C7**. From **C12** onward, the peak positions stabilize at ν_asym_(C–H) = 2918 cm^–1^ and ν_sym_(C–H) = 2850 cm^–1^, suggesting an aliphatic sublattice where additional CH_2_ units adopt *trans* conformations, consistent with a crystalline-like organization of higher paraffines.^28^

**Figure 6:**
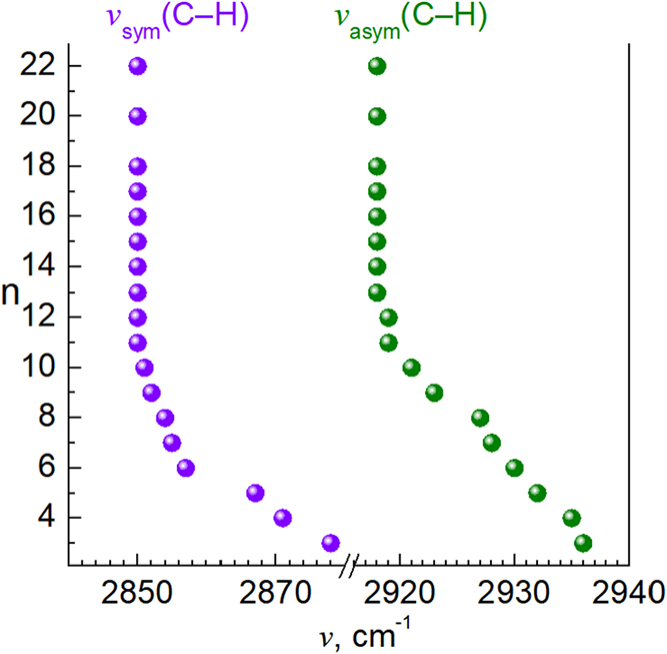
Maxima values of C–H absorption bands of **C3**–**C22**.

### Effect of Pressure on the Thermal Dependence of Magnetic Properties

To identify the effect of pressure, representative compounds with different aliphatic chain lengths have been selected and characterized by means of magnetic measurements. These are compounds **C3**, for which the short chains minimize the effect of the chain length, compounds **C15** and **C16** with long chains, whose behavior differs due to the odd–even effect, and **C8** which displays abrupt SCO without hysteresis. Figure 7 shows the effect of pressure on the thermal dependence of the χ_M_ *T* product of these compounds at the scan rate 1 K min^–1^. As pressure increases, the SCO curves move to the higher temperatures, demonstrating an increase in the *T*_1/2_ value in all cases. The transition becomes more gradual in all cases with a small divergence of the heating and cooling curves at higher temperatures.

**Figure 7:**
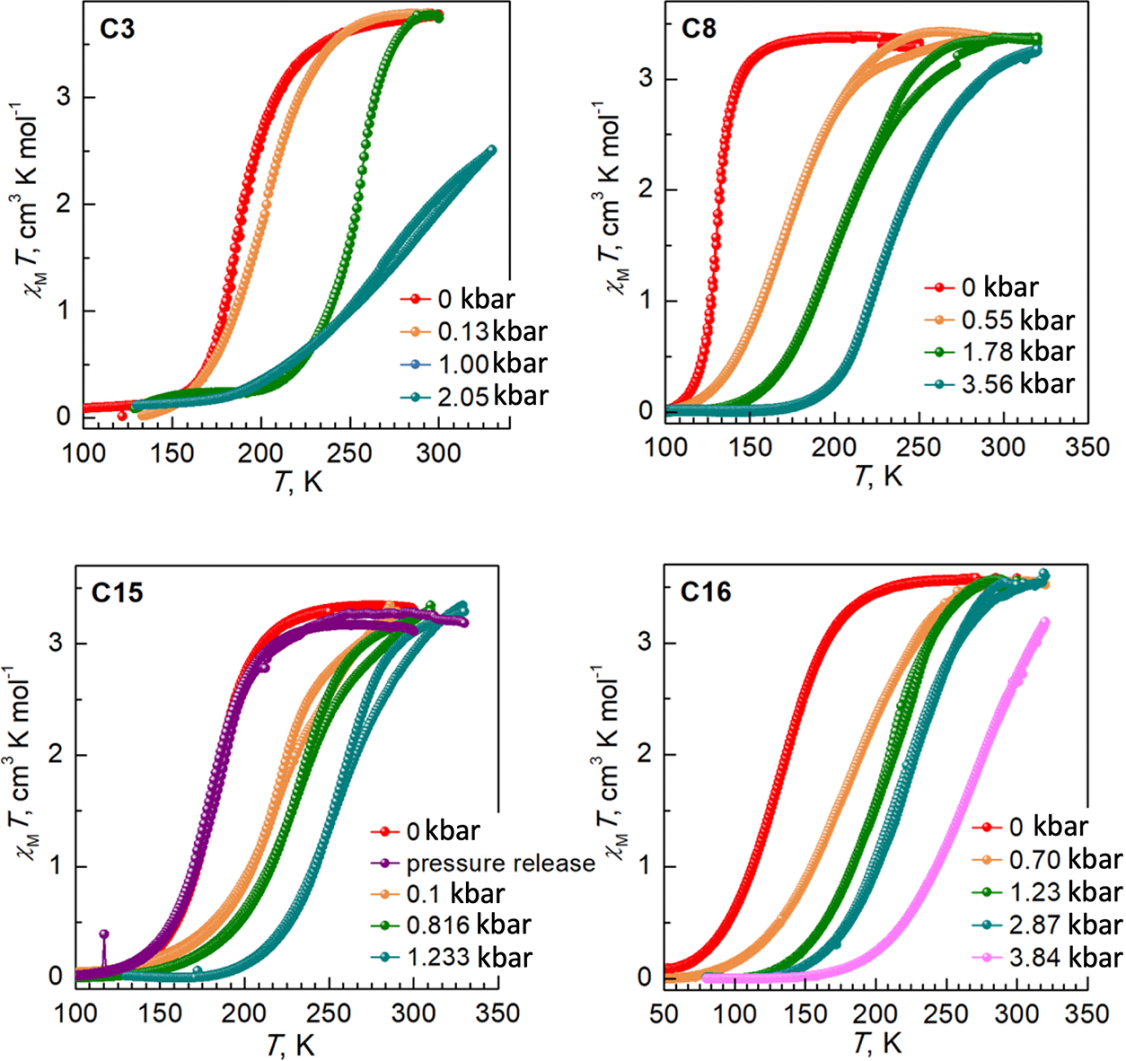
Temperature dependence of the χ_M_ *T* value under different pressures for the indicated compounds.

According to the magnetic data, the pressure sensitivity d*T*_1/2_/d*P* has a nonlinear dependence on pressure applied, first showing a strong effect which decreases upon further pressure increase (Figure 8). For **C3** the value d*T*_1/2_/d*P* is 100 K kbar^–1^ for *P* < 0.13 kbar, but decreases to 47 K kbar^–1^ at pressures above, for **C15**–386 K (*P* < 0.1 kbar) and 30 K kbar^–1^ (*P* > 0.1 kbar), for **C8**–76 K kbar^–1^ (*P* < 0.55 kbar) and 21 K kbar^–1^ (*P* > 0.55kbar), and for **C16**–62 K kbar^–1^ (*P* < 1.23 kbar) and 22 K kbar^–1^ (*P* > 0.55 kbar).

**Figure 8:**
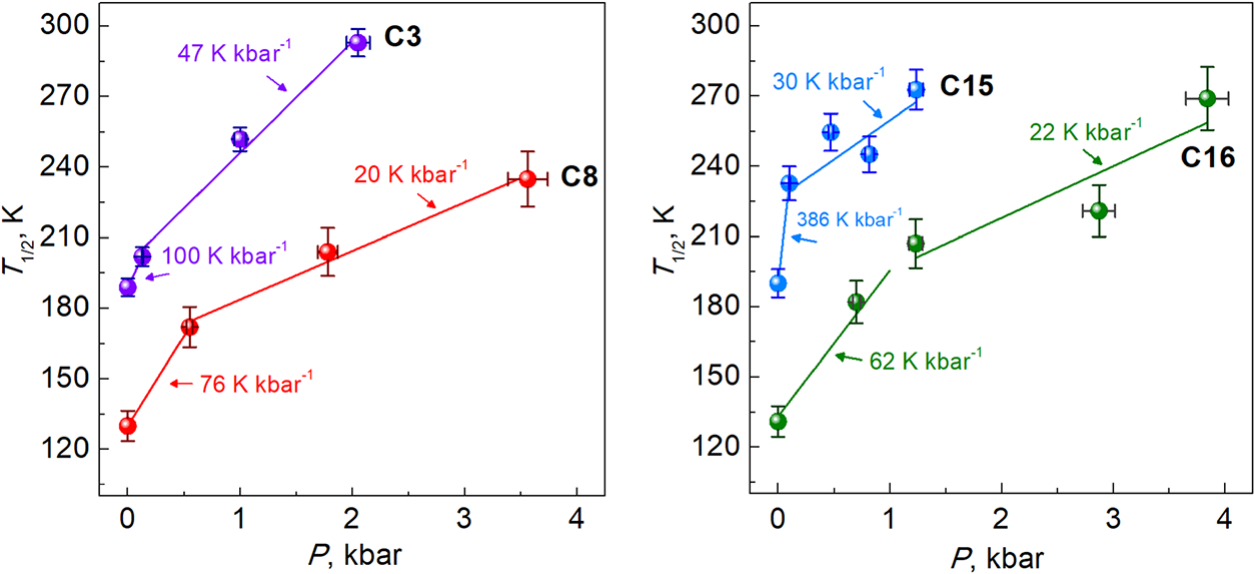
Pressure dependence of the transition temperature *T*_1/2_ on pressure for the indicated compounds.

### Pressure Effect on the IR and UV–Vis Spectroscopies at RT

The IR and UV–vis absorption spectroscopies were used to analyze the behavior of the compounds under pressure at RT (piezo-effect). Whereas IR spectroscopy is a valuable technique to analyze the response of the molecular vibration modes upon SCO,^29^ the UV–vis absorption spectroscopy follows the color change of the compounds upon SCO and studies the potential perspectives of materials to be used for optical applications.^1b,30^ Given the qualitatively similar behavior of temperature-induced SCO in the studied compounds, we have assumed that the behavior under pressure of all compounds should be essentially similar, and therefore investigated the pressure-induced SCO in only **C3** and **C15** as representative short and long chain compounds, respectively.

### IR Spectra

The changes in the vibrational modes of **C3** and **C15** recorded in the 600–3200 cm^–1^ window in the pressure range 0–13.0 kbar are displayed in Figures S9 and S10, respectively. As pressure increases, the C–H stretching vibrational peaks exhibit a hypsochromic (blue) shift due to enhanced lattice stiffness, with depressurization reversing this effect (Figures S9 and S10). The C=N stretching peak (∼1650 cm^–1^) shows a similar blue-shift, but its intensity decreases due to the direct coordination of the C=N moiety with the Fe­(II) ion transitioning from the HS to the LS state. Contrary to expectations, the C=N peak at ∼1616 cm^–1^, observed in analogous LS complexes without methyl-substituted pyridines,^13b^ is absent. The decrease in intensity of the C=N peak likely results from the enhanced d_π_–p_π_ back-bonding effect in the LS state, increasing electron delocalization and reducing bond polarity, thus lowering IR intensity. This variation in intensity of the C=N peak has been used to quantify the pressure dependence of the HS state fraction (see Figure 9) as γ_HS_ = *I*_1650_/*I*_1650max_, where *I*_1650_ is the intensity of the C=N peak at ∼1650 cm^–1^. The HS fraction decreases with increasing pressure until nearly zero at 10.0 kbar, corresponding to a full transition to the LS state. During depressurization, there is a gradual transition from LS to HS, but the pressurization and depressurization processes do not overlap completely in a pressure range 2.7 kbar ≤ *P* ≤ 8.0 kbar due to a nonhydrostatic pressure dependence of the KBr, used as pressure-transferring medium, causing errors in the fitting calculations.^31^ The errors can be neglected, therefore, we can conclude that **C3** has a pressure-induced SCO without hysteresis at RT. The derived transition pressure *P*_1/2_, where concentrations of the HS and LS molecules are equal, is 2.6 kbar.

**Figure 9:**
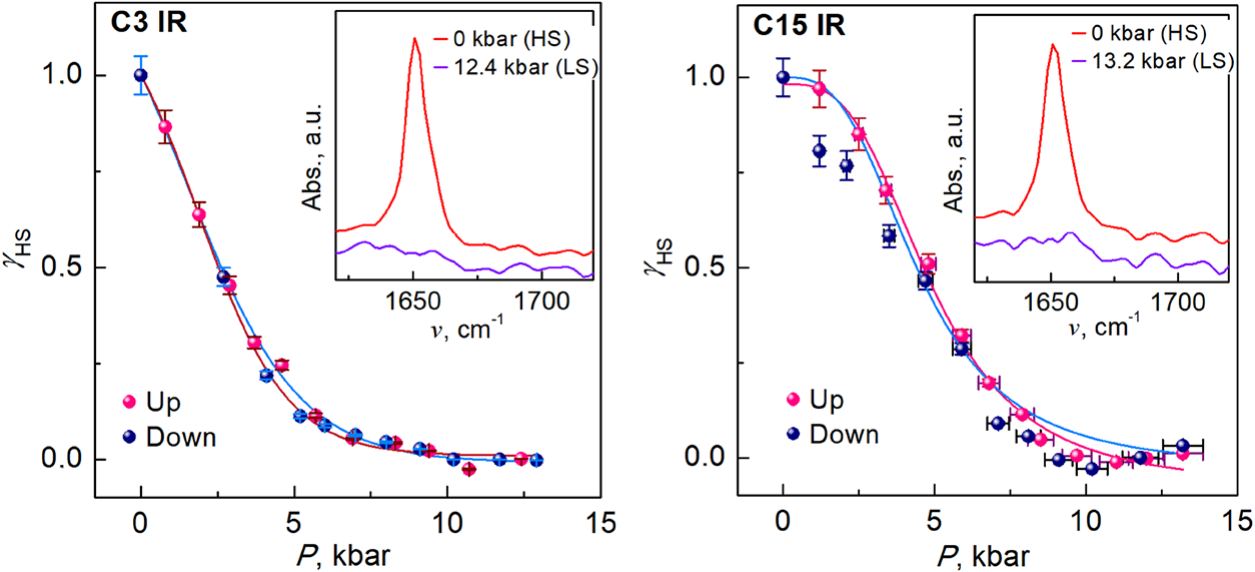
Plot γ_HS_ vs *P* for indicated compounds at RT (with IR spectra as inset).

Consistent with **C3**, the C=N vibrational band at 1650 cm^–1^ of **C15** exhibits significant variation with pressure changes. During the pressurization process, γ_HS_ gradually decreases with increasing pressure (see Figure 9), indicating that the SCO induced by pressure is complete at 10.0 kbar. The transition pressure *P*_1/2_ equals 4.7 kbar, which is significantly higher than the value of **C3**.

### UV–Vis Absorption Spectroscopy

The pressure dependence of the UV–vis absorption spectra for complexes **C3** and **C15** (0–12.0 kbar) are shown in Figures S11 and S12. Compound **C3**, being HS at ambient pressure and temperature, is characterized by a complex absorption band where a maximum can be recognized at 364 nm (peak A) with a weak shoulder at 426 nm (B), and a weaker peak at 485 nm (C) with a shoulder at 530 nm (D) (Figure 10, inset). The SCO under external pressure is accompanied by an intensification of the bands, especially of the shoulder B, which becomes the maximum at 428 nm, while other peaks exhibit a hypsochromic shift. Furthermore, an additional shoulder (E) near 650 nm becomes evident under increased pressure. Based on literature data,^1b,30^ the absorption bands are likely due to metal–ligand charge transfer (MLCT), which intensifies as the metal–ligand bond lengths shorten in the LS state at higher pressure. The additional very weak broad-band F, observed only in the HS spectrum at ∼850 nm, was attributed to the parity-forbidden ^5^ *T*_2_ → ^5^ *E* transition of the Fe^II^ ion. The absorption spectra change during pressure release, returning to their original state. Using the evolution of the intensity of the characteristic absorption peak B with pressure, we obtain the pressure dependence of the HS fraction as shown in Figure 10. The relationship between the HS fraction and pressure shows that **C3** undergoes a gradual SCO without hysteresis, with *P*_1/2_ of 4.6 kbar.

**Figure 10:**
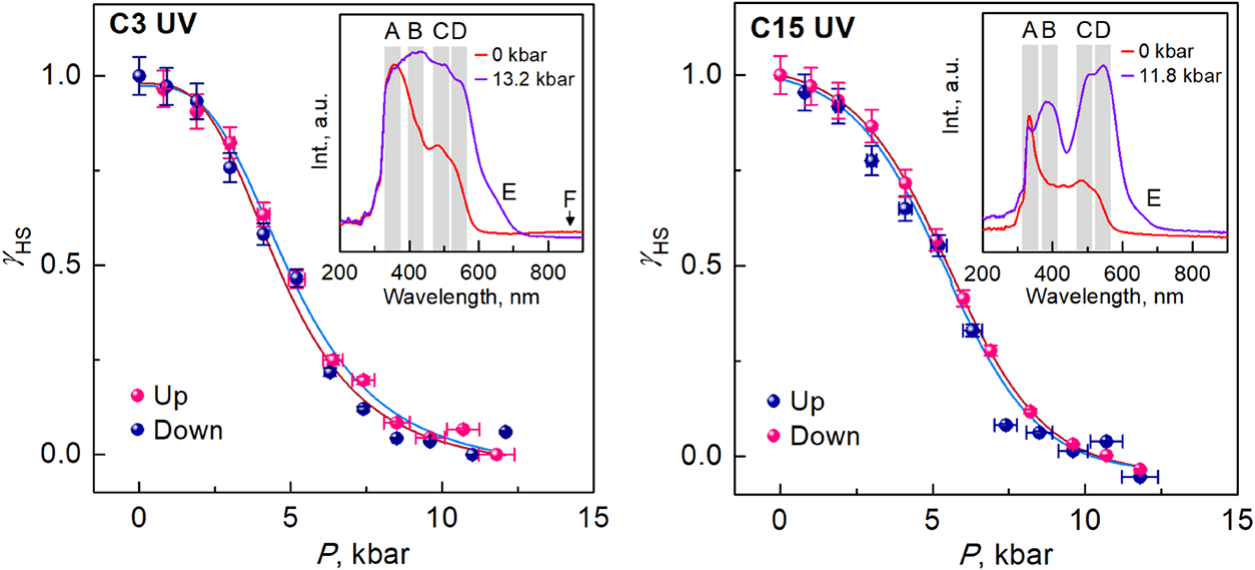
High-pressure UV–vis absorption spectra of the indicated compounds at RT.

The spectra of **C15** are very similar to those of **C3**, although the intensity of the absorption bands A and B is lower. The spectra were analyzed in the same way, and the gradual γ_HS_–*P* phase diagram without hysteresis with transition pressure equal to 5.0 kbar has been obtained (Figure 10).

A noteworthy result is that for **C15**, the transition pressure obtained from IR and optical measurements is nearly the same, whereas for **C3** it is considerably different. This difference may be attributed to the effect of the pressure transfer medium in the IR measurements, which is microcrystalline KBr. As shown by Li et al.,^31^ its influence is anomalous and can cause considerable discrepancies in measurements. However, it is not feasible to use an alternative material for IR measurements; therefore, the IR results under pressure should be considered as indicative only. Conversely, for **C15** an excellent correlation between both spectroscopic measurements suggests that grafted long aliphatic substituents serve as an additional pressure-transmitting medium, fully compensating for KBr’s nonhydrostaticity.

### Theoretical Analysis

To further understand the effect of pressure on SCO compounds, we have fit and analyzed the behavior of the HS fraction of **C3** under different pressure values based on an elastic interaction model.^23,32^ Gibbs free energy for the elastic interaction model is

1.1 G=H−TS+PV

where *H*, *T*, *S*, *P*, and V are enthalpy, temperature, entropy, pressure, and volume, respectively. The equation of state with respect to HS fractions when SCO is considered is expressed as

1.2 $$\Delta H_{\mathrm{HL}}-T\Delta S_{\mathrm{HL}}+P\Delta V_{\mathrm{HL}}+\Delta_{\mathrm{elastic}}-\Gamma +\Gamma \left(1-2\gamma_{\mathrm{HS}}\right)-N_{A}k_{B}T\ln\left(\left(1-\gamma_{\mathrm{HS}}\right)/\gamma_{\mathrm{HS}}\right)=0$$

where Δ*H*_HL_, Δ*S*_HL_, and Δ*V*_HL_ correspond to the enthalpy, entropy, and molecular volume variations, respectively, during the spin state change; Δ_elastic_ and Γ are the elastic interaction energy and the intermolecular interaction energy, respectively. The rearrangement of eq 2 yields the relationship between the high spin fraction γ_HS_ and the temperature *T*, with the expression

1.3 $$T\left(\gamma_{\mathrm{HS}}\right)=\frac{\Delta H_{\mathrm{HL}}+\left(\Delta_{\mathrm{elastic}}-\Gamma \right)+P\cdot \Delta V_{\mathrm{HL}}+\Gamma \left(1-2\gamma_{\mathrm{HS}}\right)}{\Delta S_{\mathrm{HL}}+N_{A}k_{B}\cdot \ln\left(\frac{1-\gamma_{\mathrm{HS}}}{\gamma_{\mathrm{HS}}}\right)}$$

At γ_HS_ = 1/2, eq 3 is transformed into

1.4 $$T_{1/2}=\frac{\Delta H_{\mathrm{HL}}+\left(\Delta_{\mathrm{elastic}}-\Gamma \right)+P\cdot \Delta V_{\mathrm{HL}}}{\Delta S_{\mathrm{HL}}}$$

On the assumption that Δ*H*_HL_ is independent of pressure, we can obtain the derivative of the SCO temperature with respect to pressure

1.5 $$\frac{dT_{1/2}}{dP}=+\frac{\Delta V+\frac{d\left(\Delta_{\mathrm{elastic}}-\Gamma \right)}{dP}}{\Delta V_{\mathrm{HL}}}$$

Compared to the Clausius–Clapeyron equation (*T*_1/2_/d*P* = d*V*/d*S*), eq 4 contains an additional term (Δ_elastic_ – Γ), where the difference between the elastic energy, Δ_elastic_, and the molecular interaction energy, Γ, results in a pressure-dependent deviation from linearity for the SCO temperatures. The type of the deviation is related to the sign of (Δ_elastic_ – Γ + *P*Δ*V*). The fitting of the experimental γ_HS_ vs *T* curve using eq 3 allows estimating the parameters Δ_elastic_ and Γ. The experimental Δ*H*_HL_ and Δ*S*_HL_ parameters derived from DSC measurements of **C3** are used, while Δ*V*_HL_ due to the SCO is taken from the single crystal X-ray measurements. The experimental γ_HS_ vs *T* curves at different pressures for **C3** were simulated, adjusting the values of Δ_elastic_ and Γ (see Figure S13). The pressure dependence of the resulting Δ_elastic_ and Γ parameters is shown in Figure 11 and listed in Table S6.

**Figure 11:**
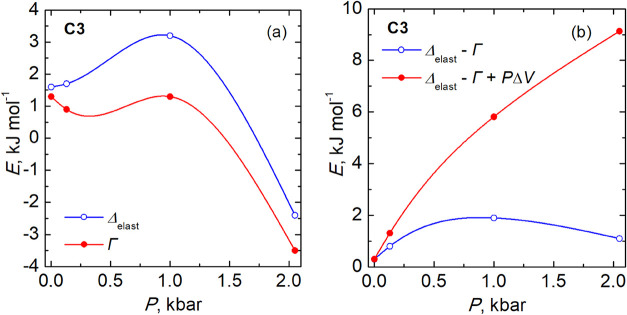
Values of the fitted parameters Δ_elastic_ and Γ (a) and their difference (b) for **C3**.

The elastic energy (Δ_elastic_ = 1.5 kJ mol^–1^) and the molecular interaction energy (Γ = 1.3 kJ mol^–1^) for **C3** at atmospheric pressure are very similar, indicating that they are equal within the experiment error. This suggests that interactions in this compound can be described within the framework of regular solution. Under a pressure of 0.13 kbar, Δ_elastic_ slightly rises to 1.7 kJ mol^–1^, while Γ drops to 0.9 kJ mol^–1^ (Figure 11a). At 1.0 kbar, Δ_elastic_ significantly increases to 3.2 kJ mol^–1^ with minimal change in Γ. At 2.05 kbar, the transition remains incomplete, and both parameters become negative: Δ_elastic_ at −2.4 kJ mol^–1^ and Γ at −3.5 kJ mol^–1^. The appearance of negative values of of Δ_elastic_ and Γ is somewhat unexpected.

In practice, when studying materials with homogeneous lattices that do not undergo structural changes under pressure, an increase in the spin transition temperature and a decrease in hysteresis are typically observed. This behavior is consistent with the Clausius–Clapeyron law, and in such materials, the elastic energy change (Δ_elastic_) is always positive. However, an increasing number of materials have recently been found to exhibit nonclassical behavior under pressure, including nonlinear changes in the transition temperature and increased hysteresis.^1e,1g^ This deviation arises from variations in the splitting energy of molecular levels and changes in intermolecular interactions under pressure. In systems exhibiting both tetragonal and trigonal lattice distortions, it becomes possible to modify the spacing between energy levels, leading to the crossing of the e_g_ and t_2g_ levels. Such level crossings can result in negative values of Δ_elastic_. A negative Δ_elastic_ may also occur in compounds where enthalpy decreases with increasing pressure. Furthermore, the emergence of a negative value for the intermolecular interaction energy (Γ) is partially attributed to changes in the energy-level spacing, although there are short-range intermolecular interactions, which are typically considered small,^1g^ and can also contribute. In certain cases, such as the material discussed here, these short-range interactions become significant, ultimately resulting in a negative Γ. To the best of our knowledge, this is the first observation of negative values for both Δ_elastic_ and Γ and the study of their behavior is on the agenda.

In summary, Δ_elastic_ and Γ show nonmonotonous behavior, increasing up to 1.0 kbar and then decreasing as pressure continues to rise. This behavior may be linked to changes in crystal structure under high pressure, order–disorder, and/or local structural distortions in the coordination polyhedron. The pressure dependence of the difference Δ_elastic_ – Γ, shown in Figure 11b, increases significantly below 1 kbar and then changes insignificantly above 1 kbar. As demonstrated in Figures 8 and 11(b), the dependence of *T*_1/2_ upon pressure exhibits a profile similar to the behavior of (Δ_elastic_ – Γ) + *P*Δ*V*, consistently observed across the studied compounds. This indicates that the observed behavior of compounds is intrinsically linked to their molecular structure.

Alongside the results of the **C3** compound, we also estimated the parameters Δ_elastic_ and Γ for compounds **C8**, **C15**, and **C16** under pressure. Reasonably good simulated γ_HS_ vs *T* curves were obtained using the experimental enthalpy Δ*H*_HL_ value of **C3** and averaged Δ*V*_HL_ of **C3** and **C7** derived from the single crystal X-ray data. Initially, Δ_elastic_ was set to zero by adjusting Δ*H* with known *T*_1/2_ and Δ*H*_HL_, the Δ*S*_HL_ was calculated as Δ*H*_HL_/*T*_1/2_. The experimental fitting provided updated values of Δ_elastic_ and Γ (see Tables S7–S9 and Figures S13–S12). The resulting Δ_elastic_ and Γ are roughly equal at ambient pressure, suggesting that the interactions can be described by the regular solutions model too. For **C3**, increasing pressure raises *T*_1/2_, while **C8**, **C15**, and **C16** show nonmonotonic behavior in Δ_elastic_ and Γ–alternating between positive and negative values–suggesting structural distortions of the octahedra (trigonal or tetragonal). This complex behavior warrants further detailed investigation and will be explored in our future work.

## Discussion and Conclusion

The primary goal of this study was to systematically investigate the influence of aliphatic chain length and external hydrostatic pressure on the SCO behavior of a series of Fe^II^ complexes. By synthesizing and characterizing a homologous series of compounds, we aimed to elucidate how a subtle and controllable chemical modification modulates the crystal packing and the intermolecular interactions and influences the transition temperature and cooperativity. Application of external pressure was explored to understand its role in tuning SCO, offering insights into the interplay between the chemical structure and the SCO response. Worth mentioning, the reinvestigation of these compounds was initiated by the discovery that using MeOH instead of EtOH as the reaction medium significantly improves the crystallization process of the title compounds. Specifically, MeOH enabled the isolation of all compounds up to **C17** in a pure homogeneous form, as evidenced by complete one-step SCO and XRD data, in contrast to the results of the previous work, where EtOH was used as the medium.^13b^ Thus, the synthesis in the same conditions provided a series of high-quality samples, enabling a detailed systematic study of the effects and pressure-dependent behaviors across the series. For longer chains, compounds **C18**, **C20** and **C22**, the problem of the solubility persists even in MeOH. Rapid precipitation from the reaction medium in the form of fine precipitate results in a significant fraction of non-SCO molecules, possibly due to high surface molecule concentration or defects. This does not influence the behavior SCO-active fraction, however, whose behavior remains consistent with those of the shorter congeners.

Although only the aliphatic chain length varies, the results show a complex, nonlinear effect that becomes more pronounced as the chain grows and dictates the SCO behavior. For short chains **C3**–**C6**, the progressive decrease in *T*_1/2_ suggests that shorter aliphatic chains destabilize LS state, likely due to decreased lattice rigidity and weaker intermolecular interactions (diminishing chemical pressure). As the chain length increases beyond **C6** and the aliphatic sublattice formation consolidates, the *T*_1/2_ values bifurcate between odd- and even-numbered chains, indicating a pronounced odd–even effect, a phenomenon known for physical properties of aliphatic compounds–density, solid and liquid crystal phase transitions, melting points *etc*.^33^ Even-numbered chain compounds (**C8**–**C22**) maintain a nearly constant *T*_1/2_, suggesting a saturation of the lattice ability to influence the SCO, possibly due to the formation of a highly ordered aliphatic sublattice. In contrast, the increasing *T*_1/2_ trend for odd-numbered chains (**C7**–**C17**) points to a restabilization of the LS state, likely arising from conformational differences in the aliphatic chains with a favorable local packing for the LS state. The cooperativity parameter Γ exhibits a pronounced odd–even variation too. For even chains, Γ steadily decreases, approaching zero by **C14**, indicative of diminishing elastic interactions. In contrast, odd chains (**C9**–**C17**) display a nonmonotonic trend, with a peak at intermediate lengths before declining. This indicates that, below a threshold length, odd chains enhance cooperativity until it is eventually outweighed by the lattice “softness”. The abrupt transitions observed in **C7** and **C8** suggest that cooperativity is maximized under conditions where the chain packing optimally balances lattice stiffness (still resembling a “normal” crystal) and flexibility of chains. As shown for **C7**, the chains are not fully fixed within an incomplete aliphatic sublattice but are long enough to show disorder in their terminal fragments, especially in less dense HS phase. However, for **C8** the cooperativity becomes attenuated and disappears upon application of a weak external pressure, becoming similar to that of the subsequent members in the series with the consolidated aliphatic sublattice, a fact that is associated with the aforementioned increase of chemical pressure.

In a broader context, the effect of the chain length change can be compared to other known methods of SCO fine-tuning, *i.e*. anion, metal or ligand dilutions.^34^ Doping SCO systems with SCO-inactive Ni^II^ or Zn^II^-based complexes disrupts the cooperativity between SCO centers, resulting in a significant downward shift of the SCO *T*_1/2_, whereas low concentrations of the dopant can fine-tune SCO behavior.^35^ In anion or ligand dilution (molecular alloys), the stoichiometry of mixed molecular components during synthesis allows optimizing desired properties with or without changing the cooperativity, as evidenced by examples of 1D and 2D coordination polymers, mononuclear complexes.^36^ The present study demonstrates that aliphatic chain length and parity act as intrinsic modulators of cooperativity without external doping.

Pressure studies show that external pressure shifts *T*_1/2_ for **C3** more significantly (47 K kbar^–1^) than for longer chain compounds. For the long chain **C15**, the effect is reduced (30 K kbar^–1^), while the sensitivity of **C8** is comparable to **C16** (∼20 K kbar^–1^), clearly demonstrating the odd–even effect in the pressure response. The nonlinear pressure sensitivity of *T*_1/2_, with d*T*_1/2_/d*P* values decreasing at higher pressures (*e.g*., 100 K kbar^–1^ to 47 K kbar^–1^ for **C3**), deviates from the linear Clausius–Clapeyron relation, indicating contributions from elastic Δ_elastic_ and intermolecular interaction energies Γ from positive demonstrating cooperative transition to negative values corresponding to anticooperative behavior. The negative values of Δ_elastic_ observed for **C3** at higher pressures (e.g., −2.4 kJ mol^–1^ at 2.05 kbar) can be attributed to pseudo-octahedral distortions under pressure, which alter the coordination environment and lattice elasticity, as seen in similar Fe^II^ systems.^1g^ However, the negative values of Γ (*e.g*., −3.5 kJ mol^–1^ for **C3** at 2.05 kbar), indicating anticooperativity, are less straightforward to rationalize. These variations might indicate how intermolecular interactions evolve under increasing pressure, with cooperativity or anticooperativity strongly influenced by the pattern and energy of these interactions within the crystal lattice.^37^ In SCO compounds, elastic interactions suggest that lattice constraints enhance cooperativity;^4^ however, this effect is not universal, as the growing influence of weak interactions may counteract this, promoting anticooperative behavior.^38^ For **C3**, **C8**, **C15**, and **C16**, pressure appears to suppress cooperative pathways by compressing the lattice, reducing the effectiveness of intermolecular interactions, which is critical for cooperative SCO.^2d,39^ Despite observed for all compounds, the suppression is particularly evident in **C3**, where Γ transit to a strongly negative value corresponding to a very gradual transition. In contrast, for longer chain compounds like **C15** and **C16**, the aliphatic sublattice mitigates the pressure-induced lattice stiffness, resulting in less pronounced anticooperativity (e.g., Γ remains closer to zero for **C16**). The odd–even effect further modulates this behavior, with odd-numbered chains (e.g., **C15**) showing slightly higher cooperativity than even-numbered chains (e.g., **C16**) due to differences in chain packing and intermolecular contacts, discussed above. These findings align with studies on related SCO systems, where pressure-induced lattice compression disrupts cooperative interactions, favoring gradual transitions.^8,9^ Further detailed structural investigations under pressure, particularly high-pressure single-crystal X-ray diffraction, would be needed to fully elucidate the structural origins of anticooperativity in the complexes.

In summary, this study reveals that SCO complexes with aliphatic substituents can be finely tuned for cooperativity and transition temperature by altering chain length. Key findings include a notable odd–even effect in *T*_1/2_ and cooperativity, influenced by nonlinear aliphatic sublattice formation and chain packing. The pressure dependence of SCO demonstrates complex lattice dynamics, with nonlinear d*T*_1/2_/d*P* trends due to competing elastic and interaction energies, showing greater pressure response in odd chains. These insights deepen understanding of structure–property relationships in aliphatic SCO systems, using chain length for structural modification.

## Experimental Section

### Synthesis of Materials

Starting reagents and solvents were obtained commercially from Aldrich and used as received. The crude alkylated picolinaldehydes was synthesized as described in ref 13b.

### General Synthetic Procedure for **C3–C22**

Crude alkylated picolinaldehyde (excess) and tris­(2-aminoethyl)­amine (41 μL, 0.27 mmol) were dissolved in methanol, then Fe­(ClO_4_)_2_·6H_2_O (100 mg, 0.27 mmol) was added, causing rapid red coloration. The mixture was stirred for 15 min and left at room temperature overnight. The resulting crystalline precipitate was filtered, washed with methanol, and air-dried. **C3:** Elemental analysis calcd. (%) for C_36_H_51_Cl_2_FeN_7_O_11_: C, 48.88; H, 5.81; N, 11.08. Found: C, 48.67; H, 5.62; N, 11.42. **C4:** Elemental analysis calcd. (%) for C_39_H_57_Cl_2_FeN_7_O_11_ Elemental Analysis: C, 50.55; H, 6.20; N, 10.58. Found: C, 50.13; H, 6.02; N, 10.23. **C5:** Elemental analysis calcd. (%) for C_42_H_63_Cl_2_FeN_7_O_11_ Elemental Analysis: C, 52.07; H, 6.55; N, 10.12. Found: C, 51.69; H, 6.32; N, 10.42. **C6:** Elemental analysis calcd. (%) for C_45_H_69_Cl_2_FeN_7_O_11_ Elemental Analysis: C, 53.47; H, 6.88; N, 9.70. Found: C, 53.23; H, 6.49; N, 9.46. **C7:** Elemental analysis calcd. (%) for C_48_H_75_Cl_2_FeN_7_O_11_ Elemental Analysis: C, 54.75; H, 7.18; N, 9.31. Found: C, 54.57; H, 7.43; N, 9.45. **C8:** Elemental analysis calcd. (%) for C_51_H_81_Cl_2_FeN_7_O_11_ Elemental Analysis: C, 55.94; H, 7.46; N, 8.95. Found: C, 56.03; H, 7.23; N, 8.99. **C9:** Elemental analysis calcd. (%) for C_54_H_87_Cl_2_FeN_7_O_11_ Elemental Analysis: C, 57.04; H, 7.71; N, 8.62. Found: C, 57.15; H, 7.56; N, 8.87. **C10:** Elemental analysis calcd. (%) for C_57_H_93_Cl_2_FeN_7_O_11_ Elemental Analysis: C, 58.06; H, 7.95; N, 8.32. Found: C, 58.01; H, 7.67; N, 8.49. **C11:** Elemental analysis calcd. (%) for C_60_H_99_Cl_2_FeN_7_O_11_ Elemental Analysis: C, 59.01; H, 8.17; N, 8.03. Found: C, 59.12; H, 8.31; N, 8.00. **C12:** Elemental analysis calcd. (%) for C_63_H_105_Cl_2_FeN_7_O_11_ Elemental Analysis: C, 59.90; H, 8.38; N, 7.76. Found: C, 59.90; H, 8.38; N, 7.76. **C13:** Elemental analysis calcd. (%) for C_66_H_111_Cl_2_FeN_7_O_11_ Elemental Analysis: C, 60.73; H, 8.57; N, 7.51. Found: C, 60.54; H, 8.31; N, 7.72. **C14:** Elemental analysis calcd. (%) for C_69_H_117_Cl_2_FeN_7_O_11_ Elemental Analysis: C, 61.50; H, 8.75; N, 7.28. Found: C, 61.76; H, 8.92; N, 7.17. **C15:** Elemental analysis calcd. (%) for C_72_H_123_Cl_2_FeN_7_O_11_ Elemental Analysis: C, 62.23; H, 8.92; N, 7.06. Found: C, 62.54; H, 9.16; N, 6.97. **C16:** Elemental analysis calcd. (%) for C_75_H_129_Cl_2_FeN_7_O_11_ Elemental Analysis: C, 62.92; H, 9.08; N, 6.85. Found: C, 63.23; H, 9.25; N, 6.63. **C17:** Elemental analysis calcd. (%) for C_78_H_135_Cl_2_FeN_7_O_11_ Elemental Analysis: C, 63.57; H, 9.23; N, 6.65. Found: C, 63.83; H, 9.46; N, 6.35. **C18:** Elemental analysis calcd. (%) for C_81_H_141_Cl_2_FeN_7_O_11_ Elemental Analysis: C, 64.18; H, 9.38; N, 6.47. Found: C, 64.46; H, 9.77; N, 6.23. **C20:** Elemental analysis calcd. (%) for C_87_H_153_Cl_2_FeN_7_O_11_ Elemental Analysis: C, 65.31; H, 9.64; N, 6.13. Found: C, 65.67; H, 9.24; N, 6.01. **C22:** Elemental analysis calcd. (%) for C_93_H_165_Cl_2_FeN_7_O_11_ Elemental Analysis: C, 66.33; H, 9.88; N, 5.82. Found: C, 66.56; H, 10.17; N, 5.64.

### Physical Methods of Characterization

#### Magnetic and Photomagnetic Measurements

Variable-temperature magnetic susceptibility data (15–20 mg) were recorded on samples at variable rates between 10 and 400 K using a Quantum Design MPMS2 SQUID magnetometer operating at 1 T magnet. The photomagnetic measurements (LIESST effect) were performed at 10 K in a commercial sample holder (Quantum Design Fiber Optic Sample Holder), wherein a quartz bucket containing *ca*. 1 mg of a sample was held against the end of a quartz fiber coupled with a laser (633 nm, 15 mW cm^–1^). After reaching the saturation of susceptibility, the sample was heated up at the rate 0.3 K min^–1^. The raw data were corrected for a diamagnetic background arising from the sample holder. The resulting magnetic signal was calibrated by scaling to match the values of the bulk sample.

#### Calorimetric Measurements

Differential scanning calorimetric (DSC) measurements were performed on a Mettler Toledo TGA/SDTA 821e under a nitrogen atmosphere with a rate of 10 K min^–1^. The raw data were analyzed with the Netzsch Proteus software with an overall accuracy of 0.2 K in the temperature and 2% in the heat flow.

#### Chemical Analysis

Elemental CHN analysis was performed after combustion at 850 °C using IR detection and gravimetry by means of a Perkin–Elmer 2400 series II device.

#### Single Crystal Crystallography

X-ray diffraction data were collected on a SuperNova (**C3**) and Nonius Kappa-CCD (**C7**) single crystal diffractometer using mirror monochromated Cu Kα radiation (λ = 1.54178 Å) and graphite monochromated Mo Kα radiation (λ = 0.71073 Å), respectively. A multiscan absorption correction was performed. The structures were solved by direct methods using SHELXS-2014 and refined by full-matrix least-squares on *F* ^2^ using SHELXL-2014.^40^ Non-hydrogen atoms were refined anisotropically and hydrogen atoms were placed in calculated positions refined using idealized geometries (riding model) and assigned fixed isotropic displacement parameters.

#### IR Spectra

IR spectra were recorded using KBr discs in the range of 4000–400 cm^–1^ with a PerkinElmer Spectrum spectrometer.

#### Mössbauer Spectra

^57^Fe Mössbauer spectra were recorded in transmission geometry on a conventional spectrometer operating in constant-acceleration mode with ^57^Co/Rh source kept at RT. The samples were sealed in a Plexiglas sample holder and mounted in a nitrogen-bath cryostat. The spectroscopic evaluations were performed with the assumption of Lorentzian line shapes by using the Recoil 1.05 Mössbauer Analysis Software (Dr. E. Lagarec). All isomeric shifts are given relative to the α-Fe at RT.

#### High Pressure Measurements

Variable temperature magnetic susceptibility under high pressure up to *P* ≈ 4 kbar were conducted on a Quantum Design MPMS-3 SQUID magnetometer at 1 T applied field over a temperature range of 50–350 K with 0.5 K min^–1^ temperature sweep rate. Pressure was applied using a piston–cylinder pressure cell fabricated from beryllium copper, pressurized externally by a hydraulic press. Silicon oil of low viscosity DC 200 was used as a pressure-transmitting medium. The pressure inside was calibrated using the pressure dependence of the superconducting transition temperature of high-purity lead.

High-pressure UV–vis absorption spectra were measured by the Ocean Optics QE65 Pro scientific research spectrometer. A diamond anvil cell, equipped with a pair of type-II diamonds, served as the pressure-generating device. A single crystal of moderate thickness was placed in a pressure chamber composed of a drilled stainless-steel gasket, and silicone oil was used as a pressure-transmitting medium. The pressure in the sample chamber was calibrated by the pressure dependence of the fluorescence emission of ruby.

High-pressure IR spectra in the range of 3500 to 600 cm^–1^ were measured on the Bruker spectrometer (VERTEX 80v). A diamond anvil cell, equipped with a pair of type-II diamonds, served as the pressure-generating device. Potassium bromide (KBr) was employed as the pressure-transmitting medium. Prior to each experiment, KBr was dried to eliminate the influence of water molecule vibrational modes.

## Supplementary Material



## Notes

Crystal data for **C3** and **C7**; Thermal variation of the crystal parameters and unit cell volume for **C3**; Selected bond lengths and angles for **C3** and **C7**; Relevant intermolecular interactions of **C6** and **C7**; Thermal dependence of Mössbauer parameters for **C3**, **C7**, **C12**–**C14**; Thermodynamic parameters, elastic energy and interaction energy for **C3**, **C8**, **C15** and **C16**; Experimental and simulated (Slichter-Drickamer model) magnetic curves for **C3**–**C20** and **C22**; ^57^Fe Mössbauer spectra for **C7**, **C12**, **C13**, and **C14** at 80 K; Thermal variation of the crystal parameters and unit cell volume for **C3**; Minimized overlay of the LS complex cations of **C3**, **C6** and **C7**; Disordered anion and fragments of the aliphatic chains of **C7** after SCO; Calculated and experimental XRPD normalized patterns for **C3** and **C7** in the HS state; Normalized powder XRD of the compounds **C3–C22** at RT; IR absorption bands C–H and C=N in the whole series; High pressure IR spectra for **C3** and **C15** at RT; High pressure UV/vis spectra for **C3** and **C15** at RT; Fitting of the ST curves under different pressures for **C3**, **C8**, **C15** and **C16**; Simulated pressure dependence of elastic energy (Δ_elastic_) and interaction parameter (Γ) for **C8**, **C15** and **C16** (PDF)
