# Supplementary material for: Aliphatic Chains and External Pressure as Tools for Fine-Tuning Spin Transition Temperature and Cooperativity
Source: Inorg Chem. 2025 Oct 9;64(48):23399–413. doi: 10.1021/acs.inorgchem.5c03403 (PMC12690579; doi:10.1021/acs.inorgchem.5c03403)

SUPPORTING INFORMATION

Aliphatic chains and external pressure as tools for fine-tuning spin transition temperature and cooperativity

Hanlin Yu,[a] Maksym Seredyuk,[b*] Kateryna Znovjyak,[b] Nikita Liedienov,[a,c*] Wei Xu,[d] Francisco Javier Valverde-Muñoz,[e] M. Carmen Muñoz,[f] Joachim Kusz,[g] Maria Książek,[g] Ruixin Li,[h] Quanjun Li,[a] Bingbing Liu,[a] Gábor Molnár,[i] Georgiy Levchenko,[a,c,j*] José Antonio Real.[k*]

(maksym.seredyuk@knu.ua; nikita.ledenev.ssp@gmail.com; g-levch@ukr.net; jose.a.real@uv.es)

[a] State Key Laboratory of High Pressure and Superhard Materials, Jilin University, 130012 Changchun, China.
[b] Department of Chemistry, Taras Shevchenko National University of Kyiv, 01601 Kyiv, Ukraine
[c] Donetsk Institute for Physics and Engineering Named after O.O. Galkin, NASU, 03028 Kyiv, Ukraine
[d] State Key Laboratory of Inorganic Synthesis and Preparative Chemistry, College of Chemistry, Jilin University, 130012 Changchun, China.
[e] CNRS IPR – UMR 6251, Univ Rennes, F-35000 Rennes, France.
[f] Departamento de Fisica Aplicada, Universitat Politècnica de València, Camino de Vera s/n, 46022 Valencia, Spain.
[g] Institute of Physics, University of Silesia, 75 Pułku Piechoty 1, 41-500 Chorzów, Poland.
[h] School of Physics Science and Information Technology, Liaocheng University, 252000 Liaocheng, China.
[i] Laboratoire de Chimie de Coordination CNRS & Université de Toulouse (UPS, INP), 205 route de Narbonne, 31077 Toulouse, France.
[j] International Center of Future Science, Jilin University, 130012 Changchun, China.
[k] Instituto de Ciencia Molecular, Departamento de Química Inorgánica, Universidad de Valencia, 46980 Paterna, Valencia, Spain.

Table of content

Table S1. Crystal data for **C3** and **C7.** S3
Table S2. Thermal variation of the crystal parameters *a*, *b*, *c*, *β* and of the unit cell volume *V* for **C3.** S3
Table S3. Selected bond lengths [Å] and angles [°] for **C3** and **C7** S4
Table S4. Comparison of intermolecular interaction of **C6** and **C7** in the LS state. S5
Table S5. Mössbauer parameters, isomer shift (IS, relative to α-iron) and quadrupole splitting (ΔE_Q), and percentage of populations in the HS and LS doublets at different temperatures for the indicated compounds. S6
Table S6. Thermodynamic parameters, elastic energy, and interaction parameter of the **C3** under pressure. S6
Table S7. Thermodynamic parameters, elastic energy, and interaction parameter of the **C8** under pressure. S6
Table S8. Thermodynamic parameters, elastic energy, and interaction parameter of the **C15** under pressure. S6
Table S9. Thermodynamic parameters, elastic energy, and interaction parameter of the **C16** under pressure. S6
Figure S1. Experimental magnetic curves and the curves simulated by the Slichter-Drickamer model of the indicated compounds. S7
Figure S2. ^{57}Fe Mössbauer spectra of the indicated compounds at 80 K. S8
Figure S3. Thermal variation of the crystal parameters *a*, *b*, *c*, *β* and of the unit cell volume *V* for **C3**. S9
Figure S4. The minimized overlay of the LS complex cations of **C3** (blue), **C6** (orange), and **C7** (violet). S9
Figure S5. Disordered anion and fragments of the aliphatic chains of **C7** highlighted in cyan after SCO. S10
Figure S6. Calculated (red) and experimental XRPD normalized patterns for **C3** (HS) and **C7** (HS) displaying the dominant very intense [100] peak below $2\theta = 7°$. S10
Figure S7. Normalized powder XRD of the compounds at RT. S11
Figure S8. IR absorption bands C–H and C=N in the series. S12
Figure S9. High-pressure IR spectra of **C3** at RT. S12
Figure S10. High-pressure IR spectra of **C15** at RT. S13
Figure S11. High-pressure UV/vis spectra of **C3** at RT. S13
Figure S12. High-pressure UV/vis spectra of **C15** at RT. S14
Figure S13. Fitting (black curve) of the SCO curves under different pressures for the indicated compounds. S15
Figure S14. Left: Variation of elastic energy ($\Delta_{elastic}$) and interaction parameter (Γ) with pressure, derived from simulation. Right: Difference between $\Delta_{elastic}$ and Γ, along with the value of ($\Delta_{elastic} - \Gamma$) + $P\Delta V$, under varying pressures for indicated compounds. S16

Table S1. Crystal data for **C3** and **C7**.

Temperature	**C3** 100 K	**C3** 250 K	**C7** 120 K	**C7** 250K
Empirical formula	$C_{36}H_{51}N_7O_{11}Cl_2Fe$		$C_{48}H_{75}N_7O_{11}Cl_2Fe$	
*M*r	884.59		1052.90	
Crystal system	monoclinic		monoclinic	
Space group	$P2_1/c$		$P2_1/c$	
a (Å)	13.7894(3)	14.2251(3)	18.9549(10)	19.1336(13)
b (Å)	19.7109(4)	20.0893(4)	19.3462(8)	19.7598(10)
c (Å)	14.9301(4)	15.1098(3)	14.8467(7)	15.0557(8)
β (°)	90.578(2)	90.078(6)	103.160(5)	98.460(5)
V (Å^3)	4057.8(2)	4318.0(2)	5301.4(4)	5630.3(6)
Z	4		4	
D_c (mg cm^{-3})	1.448	1.361	1.319	1.242
F(000)	1856		2240	
μ (Cu-K$_\alpha$) (mm^{-1})	4.762	4.475		
μ (Mo-K$_\alpha$) (mm^{-1})			0.448	0.422
Crystal size (mm)	0.02x0.07x0.18		0.01x0.03x0.05	
No. of total reflections	7181	7598	9311	9890
No. of reflections [$I>2\sigma(I)$]	6407	5876	4110	2599
R [$I>2\sigma(I)$]	0.0816	0.0621	0.0871	0.0927
wR [$I>2\sigma(I)$]	0.1954	0.1631	0.1305	0.1611
S	1.014	1.038	1.022	1.027

$R_1 = \Sigma\,||F_o| - |Fc||/\Sigma|F_o|$; $wR = [\Sigma\,[w(F_o^2 - Fc^2)^2]/\Sigma\,[w(F_o^2)^2]]^{1/2}$;
$w = 1/\,[\sigma^2(F_o^2) + (m\,P)^2 + n\,P]$, where $P = (F_o^2 + 2F_c^2)/3$;
m(**C3**) = 0.0861 (100 K) and 0.0913 (250 K); n(**C3**) = 24.5496 (100K) and 2.8993 (250 K);
m(**C7**) = 0.0233 (120 K) and 0.0490 (250 K); n(**C7**) = 0.0000 (120K) and 0.0000 (250 K).

Table S2. Thermal variation of the crystal parameters *a*, *b*, *c*, *β* and of the unit cell volume *V* for **C3**.

***T*, K**	***a*, Å**	***b*, Å**	***c*, Å**	**β, °**	***V*, Å^3**
260	14.2480	20.0807	15.1200	90.119	4326.0
240	14.2018	20.0621	15.0953	90.036	4300.9
220	14.1484	20.0317	15.0705	89.903	4271.2
200	14.0817	19.9752	15.0424	89.720	4231.1
180	14.0039	19.8813	15.0097	89.627	4178.9
160	13.9332	19.791	14.9716	89.854	4128.4
140	13.8873	19.746	14.9492	89.982	4099.4
120	13.8572	19.7163	14.9322	90.021	4079.7
100	13.8273	19.6979	14.9200	89.990	4063.7

Table S3. Selected bond lengths [Å] and angles [°] for **C3** and **C7**

	**C3** 100 K	**C3** 250 K		**C7** 120 K	**C7** 250K
Fe-N(11)	1.957(4)	2.125(4)	Fe-N(1)	2.069(5)	2.277(8)
Fe-N(12)	2.078(4)	2.303(4)	Fe-N(2)	1.950(5)	2.137(7)
Fe-N(21)	1.952(4)	2.122(4)	Fe-N(3)	2.087(5)	2.329(7)
Fe-N(22)	2.101(4)	2.334(4)	Fe-N(4)	1.965(5)	2.114(8)
Fe-N(31)	1.948(4)	2.113(4)	Fe-N(5)	2.084(5)	2.343(6)
Fe-N(32)	2.086(4)	2.308(4)	Fe-N(6)	1.932(5)	2.119(7)
N(11)-Fe-N(12)	80.9(2)	75.3(2)	N(1)-Fe-N(2)	81.2(2)	75.7(3)
N(11)-Fe-N(21)	93.0(2)	99.8(2)	N(1)-Fe-N(3)	101.2(2)	101.8(3)
N(11)-Fe-N(22)	84.1(2)	85.71(14)	N(1)-Fe-N(4)	174.0(2)	174.7(3)
N(11)-Fe-N(31)	93.9(2)	99.5(2)	N(1)-Fe-N(5)	101.8(2)	102.3(3)
N(11)-Fe-N(32)	173.9(2)	173.94(14)	N(1)-Fe-N(6)	84.5(2)	86.8(3)
N(12)-Fe-N(21)	173.5(2)	174.9(2)	N(2)-Fe-N(3)	85.4(2)	87.0(2)
N(12)-Fe-N(22)	100.3(2)	103.02(13)	N(2)-Fe-N(4)	93.7(2)	99.3(3)
N(12)-Fe-N(31)	84.9(2)	86.50(14)	N(2)-Fe-N(5)	174.0(2)	173.8(3)
N(12)-Fe-N(32)	101.3(2)	100.54(13)	N(2)-Fe-N(6)	94.0(2)	99.4(3)
N(21)-Fe-N(22)	81.1(2)	74.9(2)	N(3)-Fe-N(4)	81.5(2)	75.9(3)
N(21)-Fe-N(31)	93.5(2)	95.9(2)	N(3)-Fe-N(5)	99.0(2)	99.2(3)
N(21)-Fe-N(32)	84.6(2)	84.4(2)	N(3)-Fe-N(6)	174.0(2)	170.5(3)
N(22)-Fe-N(31)	174.1(2)	170.11(14)	N(4)-Fe-N(5)	83.0(2)	82.9(2)
N(22)-Fe-N(32)	100.9(2)	99.63(12)	N(4)-Fe-N(6)	92.7(2)	95.9(3)
N(31)-Fe-N(32)	80.7(2)	75.65(14)	N(5)-Fe-N(6)	81.3(2)	74.6(3)

Table S4. Comparison of intermolecular interaction of **C6** and **C7** in the LS state.

C6 90K

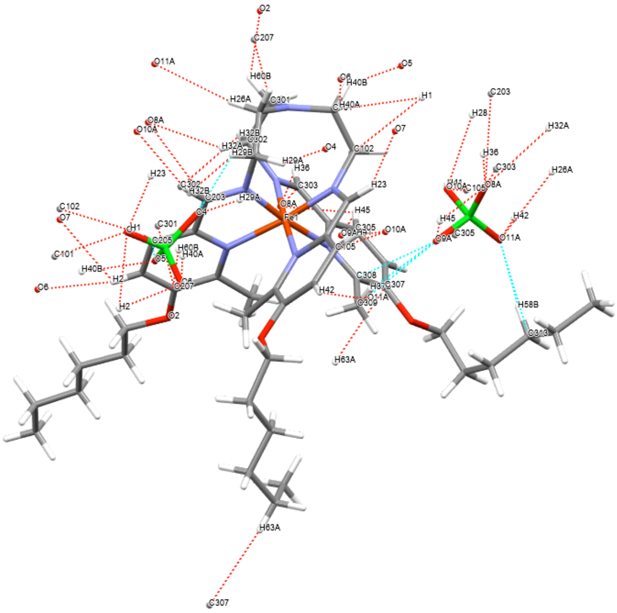

C7 120 K

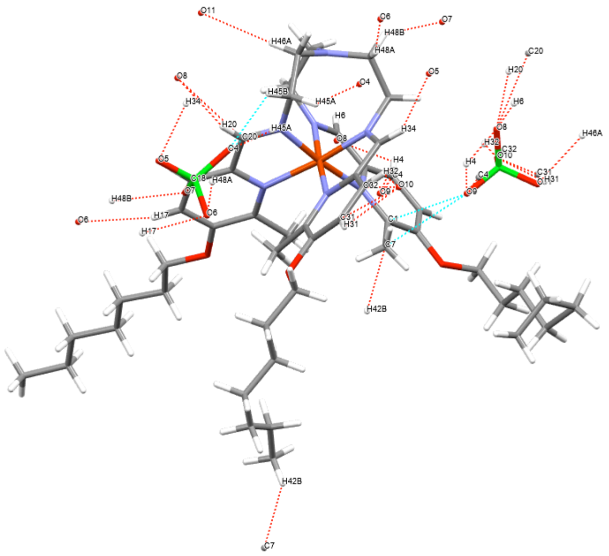

Short contact	Length, Å
O11A···H26A	2.684
O10A···H28	2.471
O8A···C203	3.135
O8A···H32A	2.706
O11A···C313	3.154
O11A···H58B	2.302
O9A···C309	3.159
O9A···H37B	2.555
O9A···C308	3.119
O9A···C305	3.142
O9A···H45	2.465
O8A···C303	3.202
O8A···H36	2.606
O8A···H45	2.693
O11A···H42	2.574
O10A···C105	3.175
O10A···H41	2.393
H1···C102	2.823
H1···C101	2.807
O2···H60B	2.712
C207···C301	3.347
C302···H32B	2.839
C307···H63A	2.824
C205···O5	3.135
C203···O5	3.119
H29B···O4	2.587
H40B···O5	2.536
H23···O7	2.606
H29A···O4	2.515
H40A···O6	2.520
H2···O7	2.718
H2···O6	2.400

Short contact	Length, Å
C7···H42B	2.807
C18···O7	3.139
C20···O7	3.191
H45B···O4	2.706
H48B···O7	2.656
H34···O5	2.658
H45A···O4	2.587
H48A···O6	2.576
H17···O6	2.505
C20···O8	3.043
H20···O8	2.629
H46A···O11	2.708
C1···O9	3.151
C7···O9	3.163
C31···O10	3.205
H31···O10	2.638
C32···O10	3.171
H32···O10	2.548
C4···O9	3.174
H4···O8	2.547
H4···O9	2.567
H6···O8	2.608

Table S5. Mössbauer parameters, isomer shift (IS, relative to α-iron) and quadrupole splitting (ΔE_Q), and percentage of populations in the HS and LS doublets at different temperatures for the indicated compounds.

Compound	*T*, K	IS, mm s^{-1}	ΔE_Q, mm s^{-1}	A, %
**C3**	80	0.47(1)	0.29(0)	100
	293	0.91(1)	1.29(2)	100
**C7**	80	0.48(1)	0.28(1)	100
**C12**	80	0.46(1)	0.25(1)	100
**C13**	80	0.47(1)	0.29(1)	100
**C14**	80	0.46(1)	0.27(1)	100

Table S6. Thermodynamic parameters, elastic energy, and interaction parameter of the **C3** under pressure.

P, kbar	Δ*H*, kJ mol^{-1}	Δ*S*, J mol^{-1} K^{-1}	$T_{1/2}$, K	Δ*V*, Å^3	$\Delta_{elastic}$, J mol^{-1}	*Γ*, J mol^{-1}
0.001	15.6	84.2	191	65.05	1600	1300
0.13	15.6	84.2	202	65.05	1700	900
1.00	15.6	84.2	252	65.05	3200	1300
2.05	15.6	84.2	293	65.05	-2400	-3500

Table S7. Thermodynamic parameters, elastic energy, and interaction parameter of the **C8** under pressure.

P, kbar	Δ*H*, kJ mol^{-1}	Δ*S*, J mol^{-1} K^{-1}	$T_{1/2}$, K	Δ*V*, Å^3	$\Delta_{elastic}$, J mol^{-1}	*Γ*, J mol^{-1}
0.001	12.5	95.42	131	73.64	1450	1450
0.55	12.5	95.42	173	73.64	650	-900
1.78	12.5	95.42	205	73.64	-1550	-800
3.56	12.5	95.42	237	73.64	-5650	0

Table S8. Thermodynamic parameters, elastic energy, and interaction parameter of the **C15** under pressure.

P, kbar	Δ*H*, kJ mol^{-1}	Δ*S*, J mol^{-1} K^{-1}	$T_{1/2}$, K	Δ*V*, Å^3	$\Delta_{elastic}$, J mol^{-1}	*Γ*, J mol^{-1}
0.001	18.0	99.448	181	73.64	700	700
0.10	18.0	99.448	220	73.64	3300	-200
0.816	18.0	99.448	231	73.64	1400	0
1.233	18.0	99.448	256	73.64	2500	500

Table S9. Thermodynamic parameters, elastic energy, and interaction parameter of the **C16** under pressure.

P, kbar	Δ*H*, kJ mol^{-1}	Δ*S*, J mol^{-1} K^{-1}	$T_{1/2}$, K	Δ*V*, Å^3	$\Delta_{elastic}$, J mol^{-1}	*Γ*, J mol^{-1}
0.001	11.2	84.848	132	73.64	-1300	-1300
0.70	11.2	84.848	181	73.64	-600	-1700
1.23	11.2	84.848	206	73.64	200	-600
2.87	11.2	84.848	222	73.64	-5500	-300
3.84	11.2	84.848	269	73.64	-5400	0

Figure S1. Experimental magnetic curves and the curves simulated by the Slichter-Drickamer model of the indicated compounds.

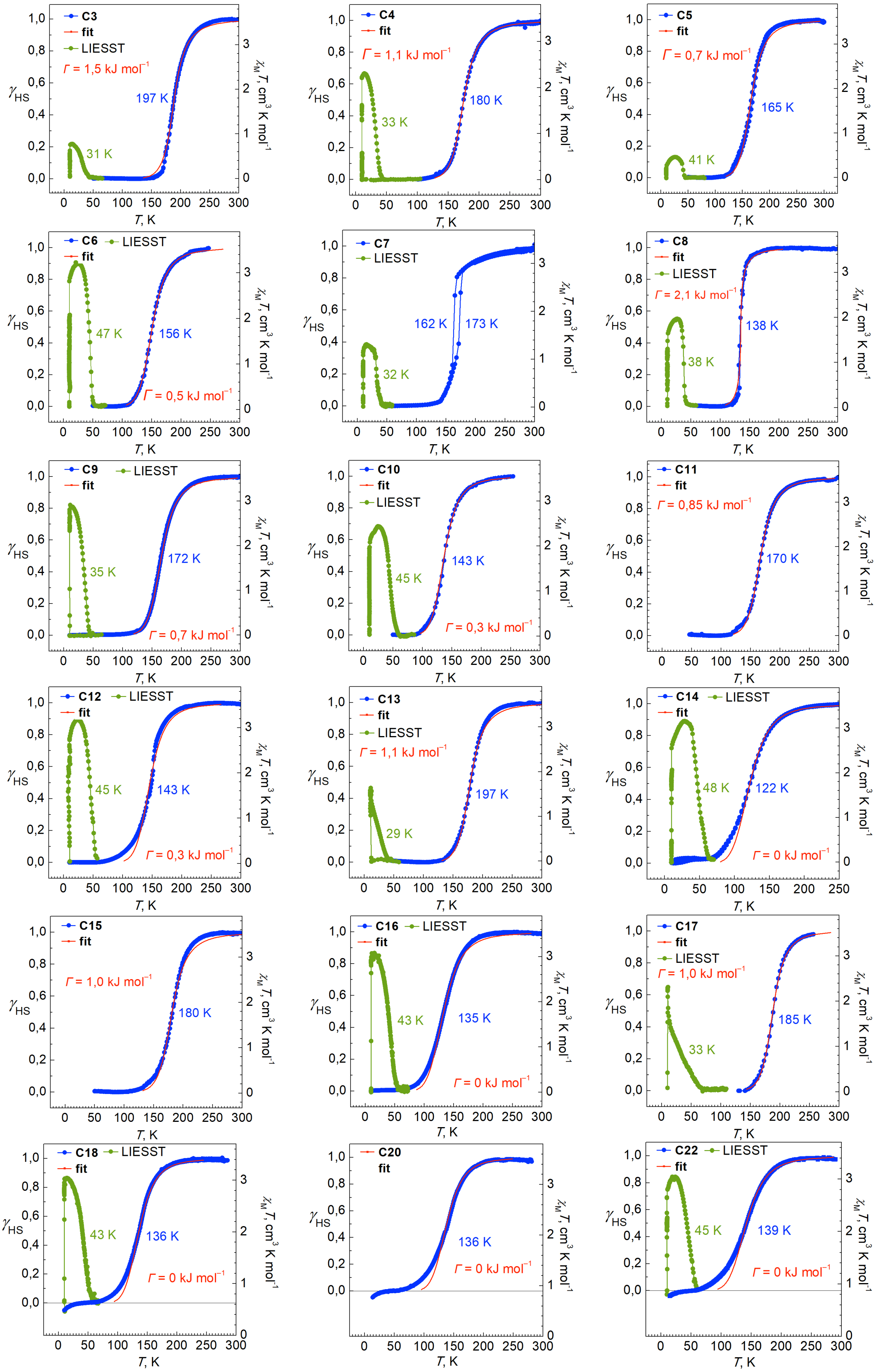

Figure S2. ^{57}Fe Mössbauer spectra of the indicated compounds at 80 K.

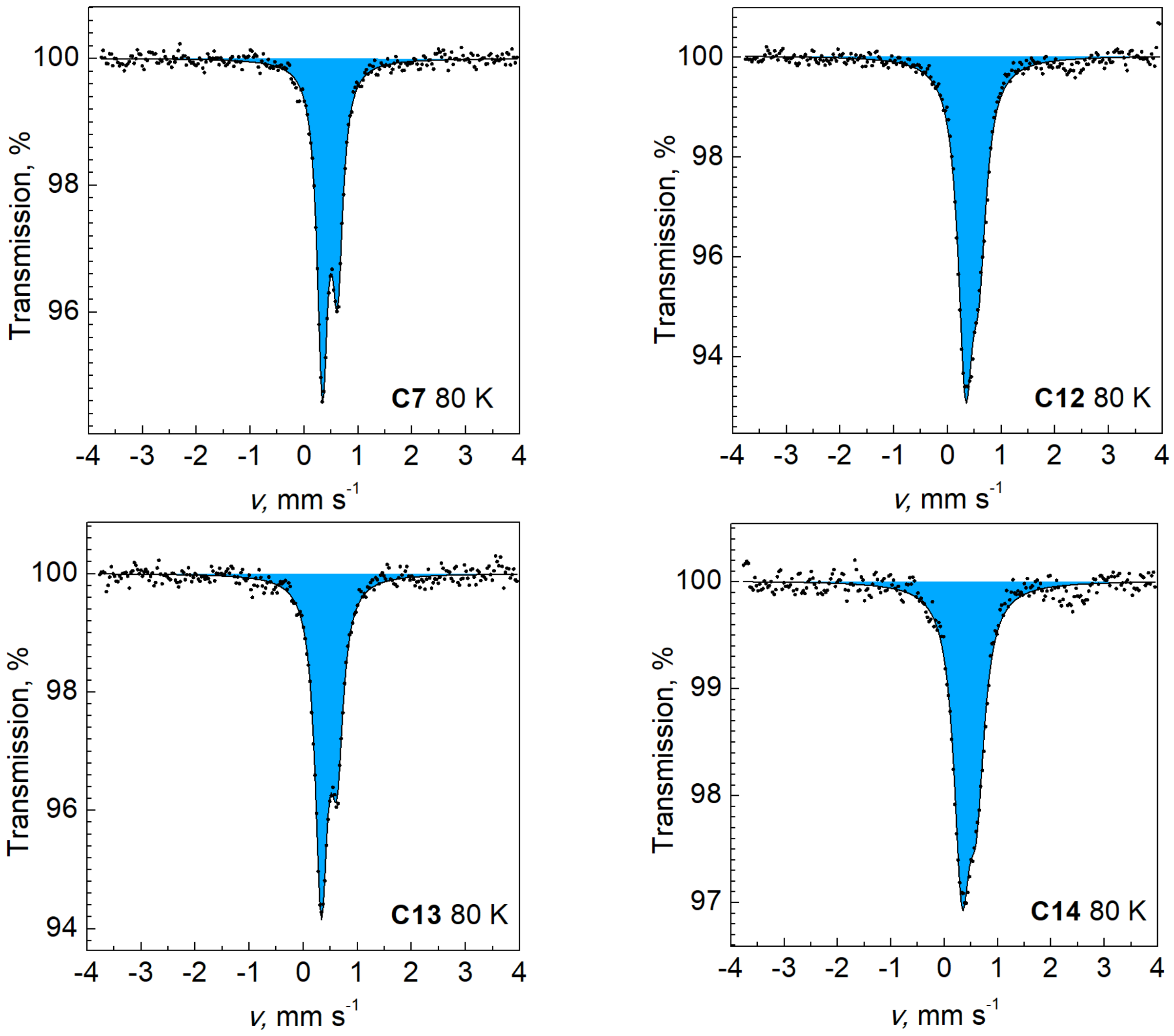

Figure S3. Thermal variation of the crystal parameters *a*, *b*, *c*, *β* and of the unit cell volume *V* for **C3**.

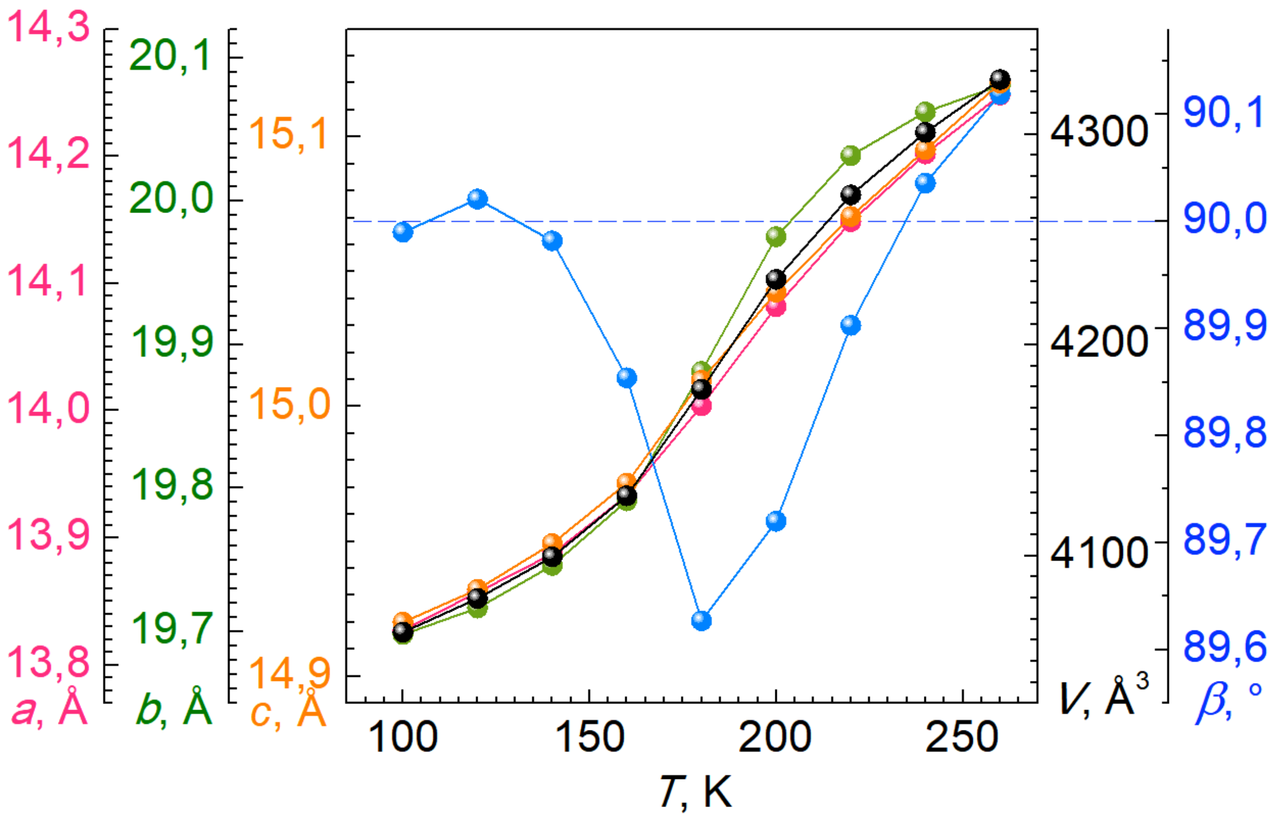

Figure S4. The minimized overlay of the LS complex cations of **C3** (blue), **C6** (orange), and **C7** (violet).

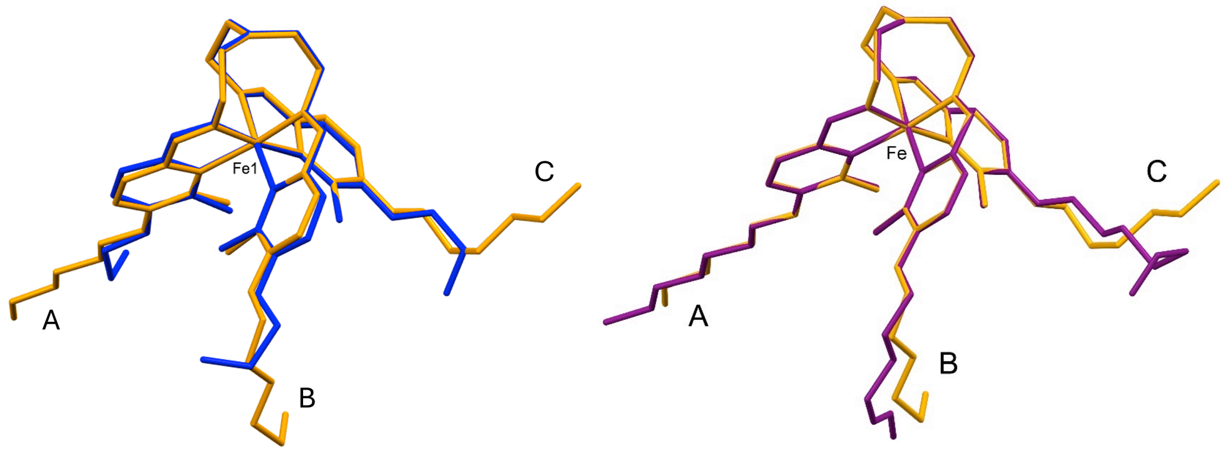

Figure S5. Disordered anion and fragments of the aliphatic chains of **C7** highlighted in cyan after SCO.

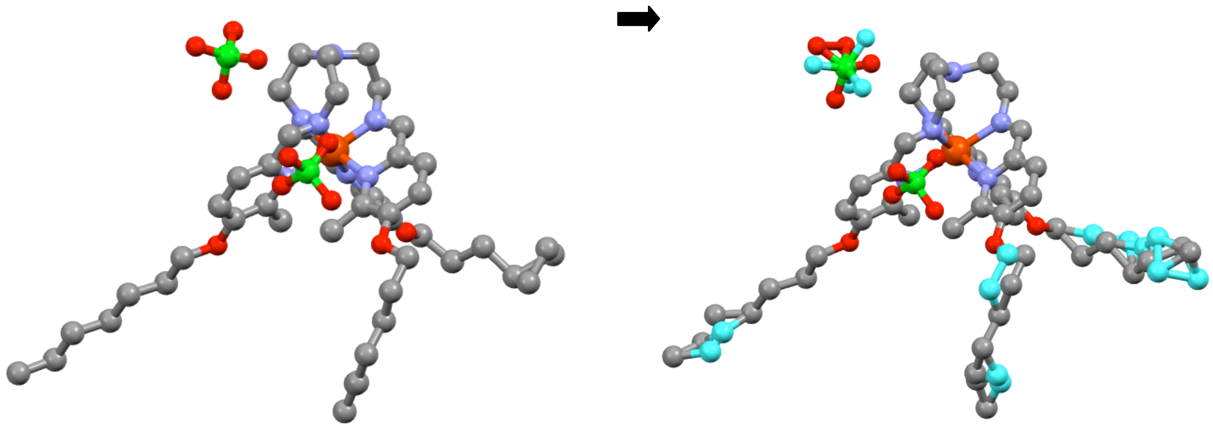

LS at 120 K   **HS** at 250 K

Figure S6. Calculated (red) and experimental XRPD normalized patterns for **C3** (HS) and **C7** (HS) displaying the dominant very intense [100] peak below $2\theta = 7°$.

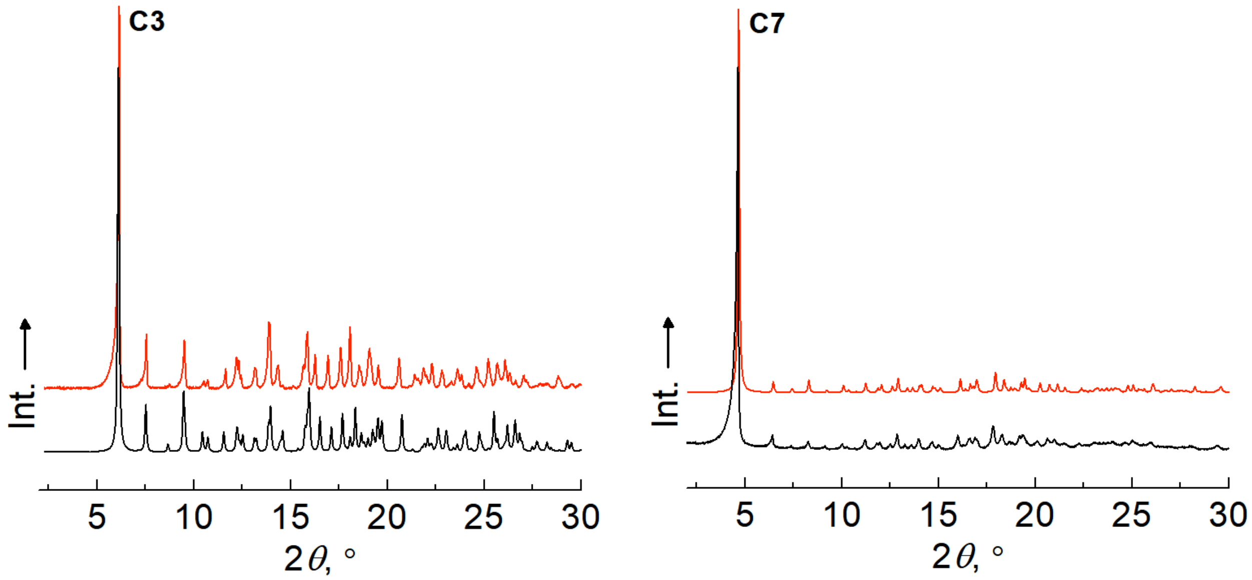

Figure S7. Normalized powder XRD of the compounds at RT.

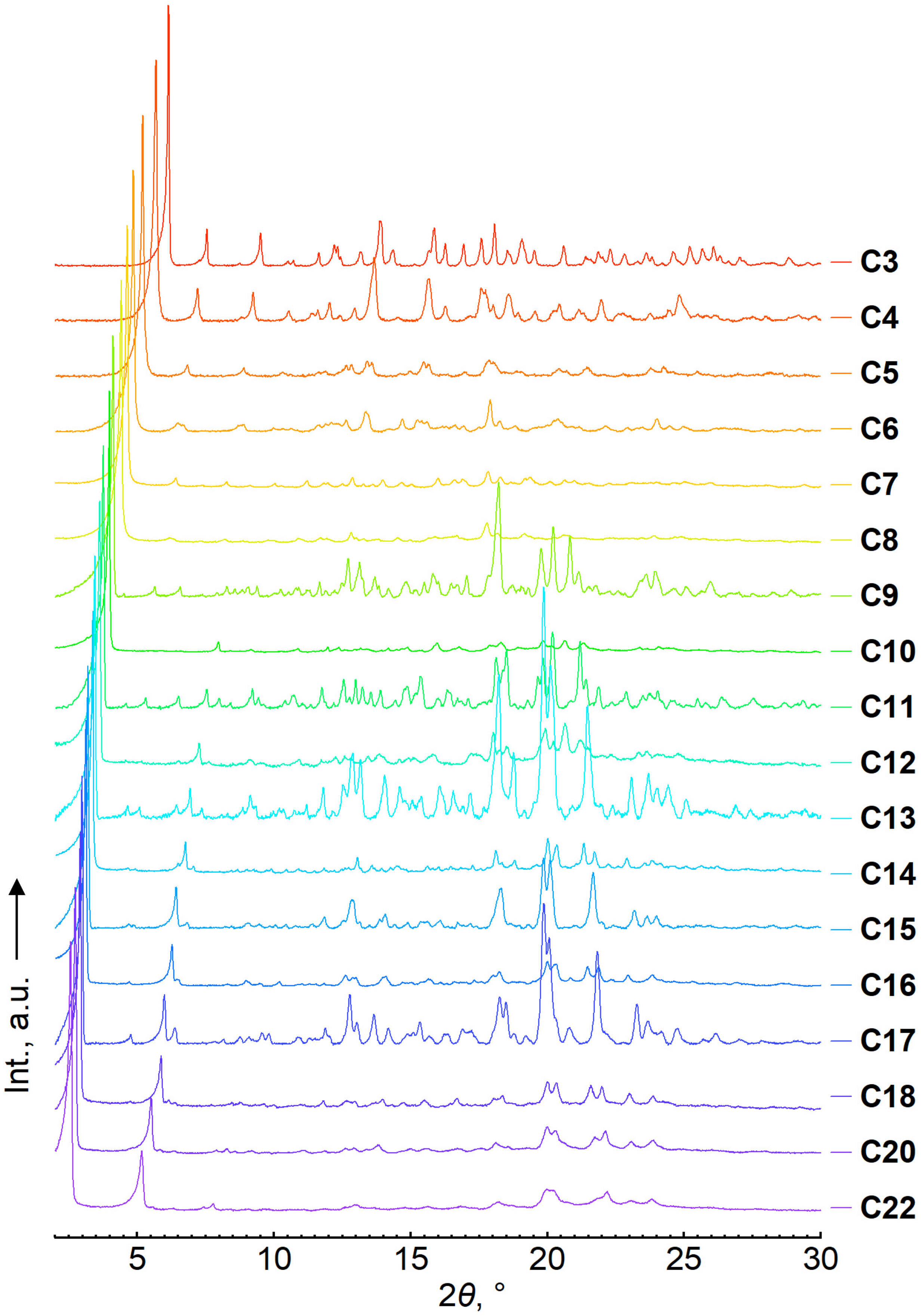

Figure S8. IR absorption bands C–H and C=N in the series.

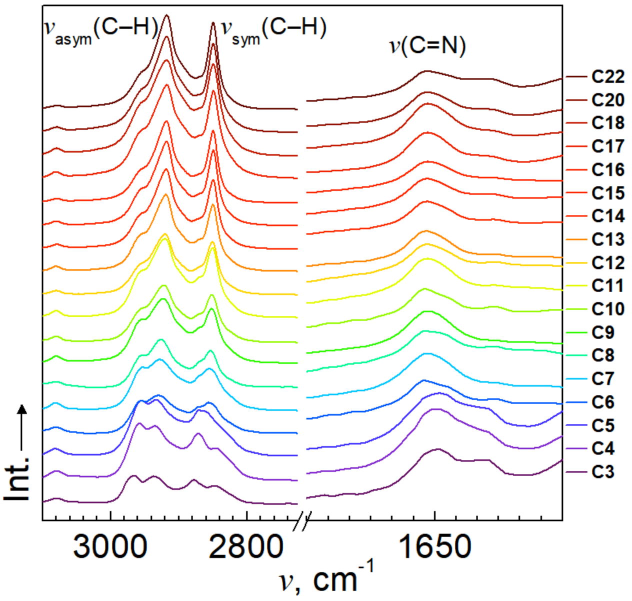

Figure S9. High-pressure IR spectra of **C3** at RT.

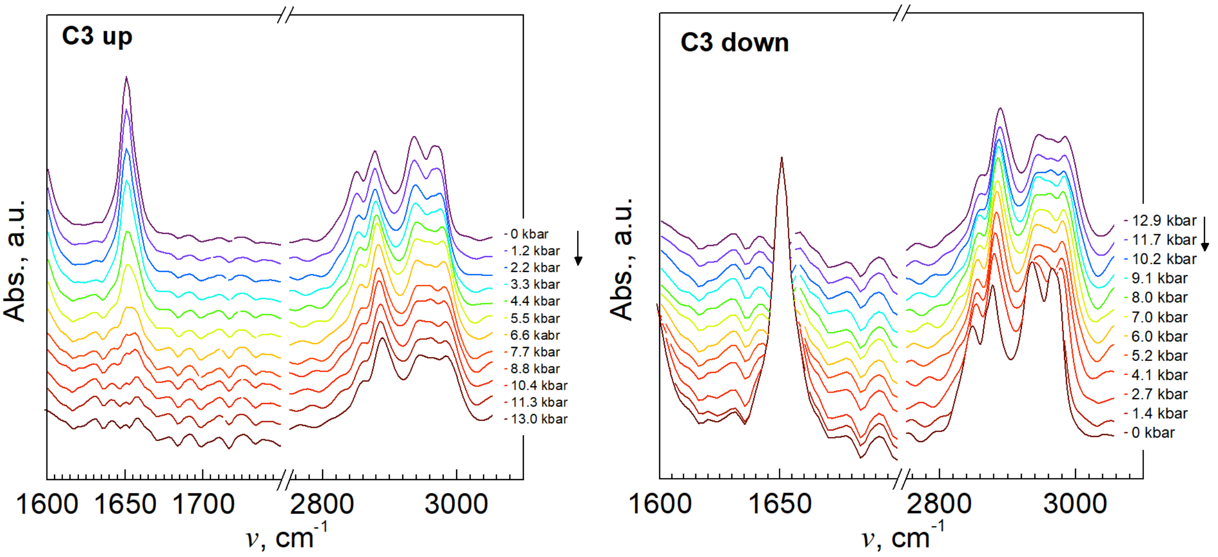

Figure S10. High-pressure IR spectra of **C15** at RT.

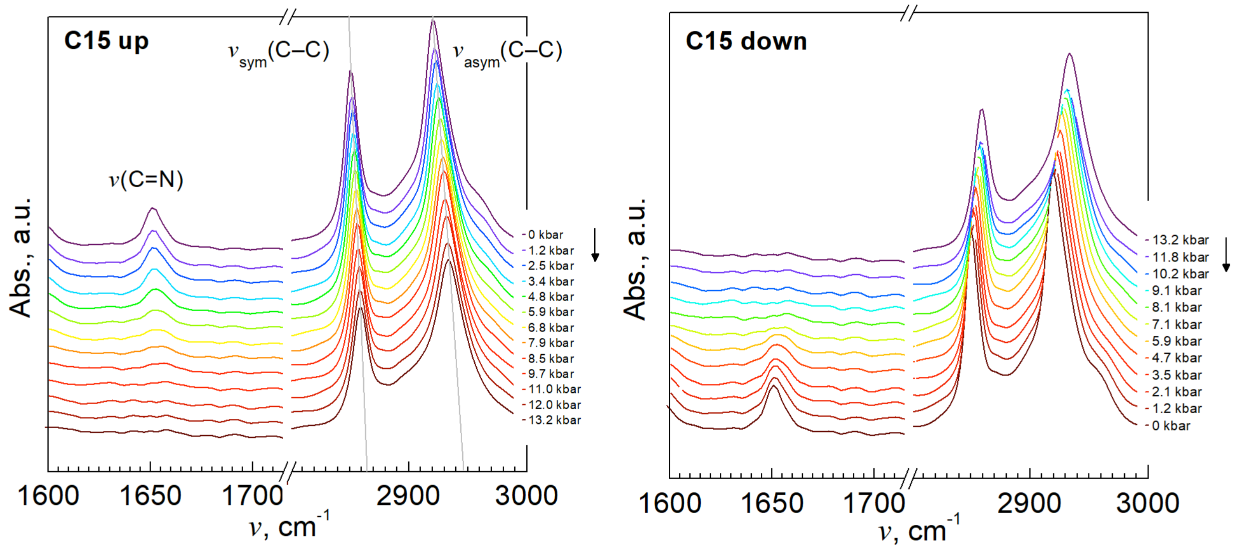

Figure S11. High-pressure UV/vis spectra of **C3** at RT.

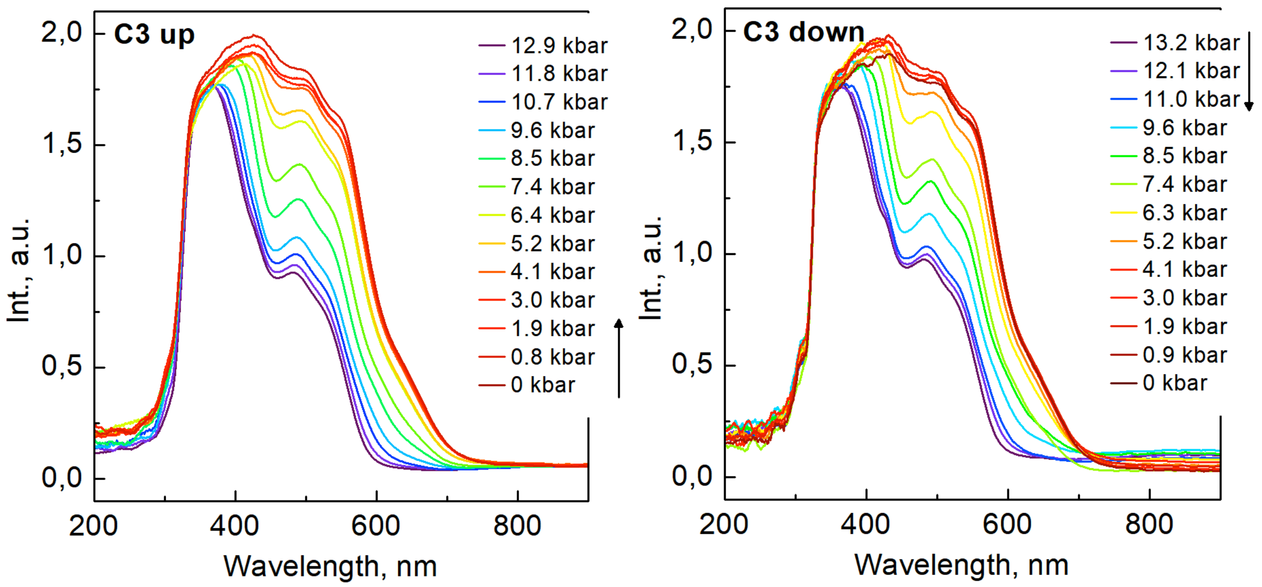

Figure S12. High-pressure UV/vis spectra of **C15** at RT.

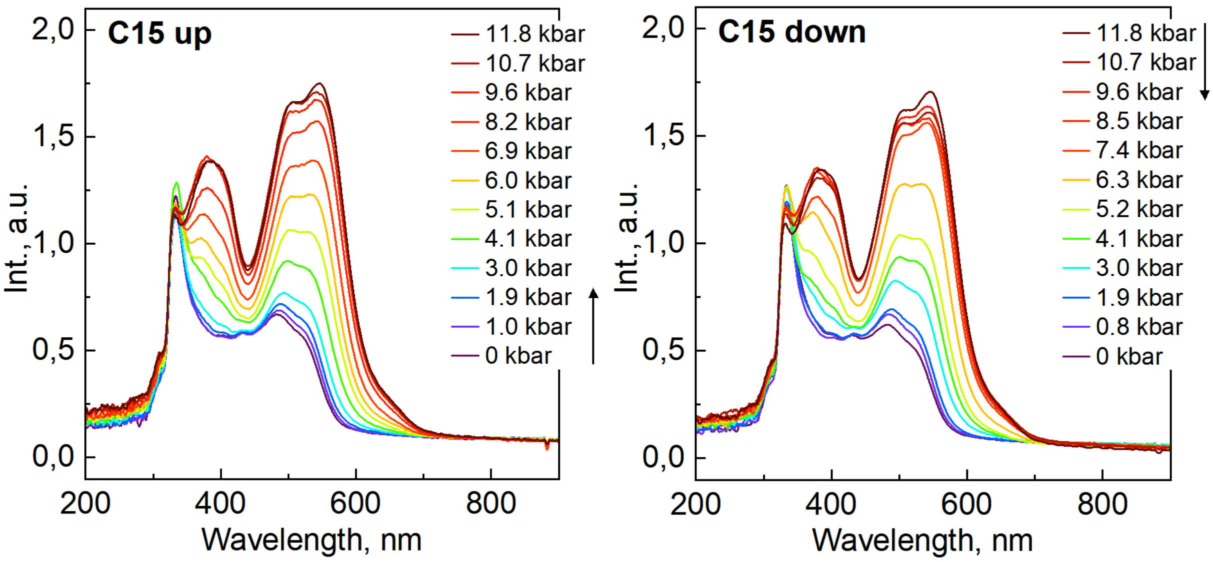

Figure S13. Fitting (black curve) of the SCO curves under different pressures for the indicated compounds.

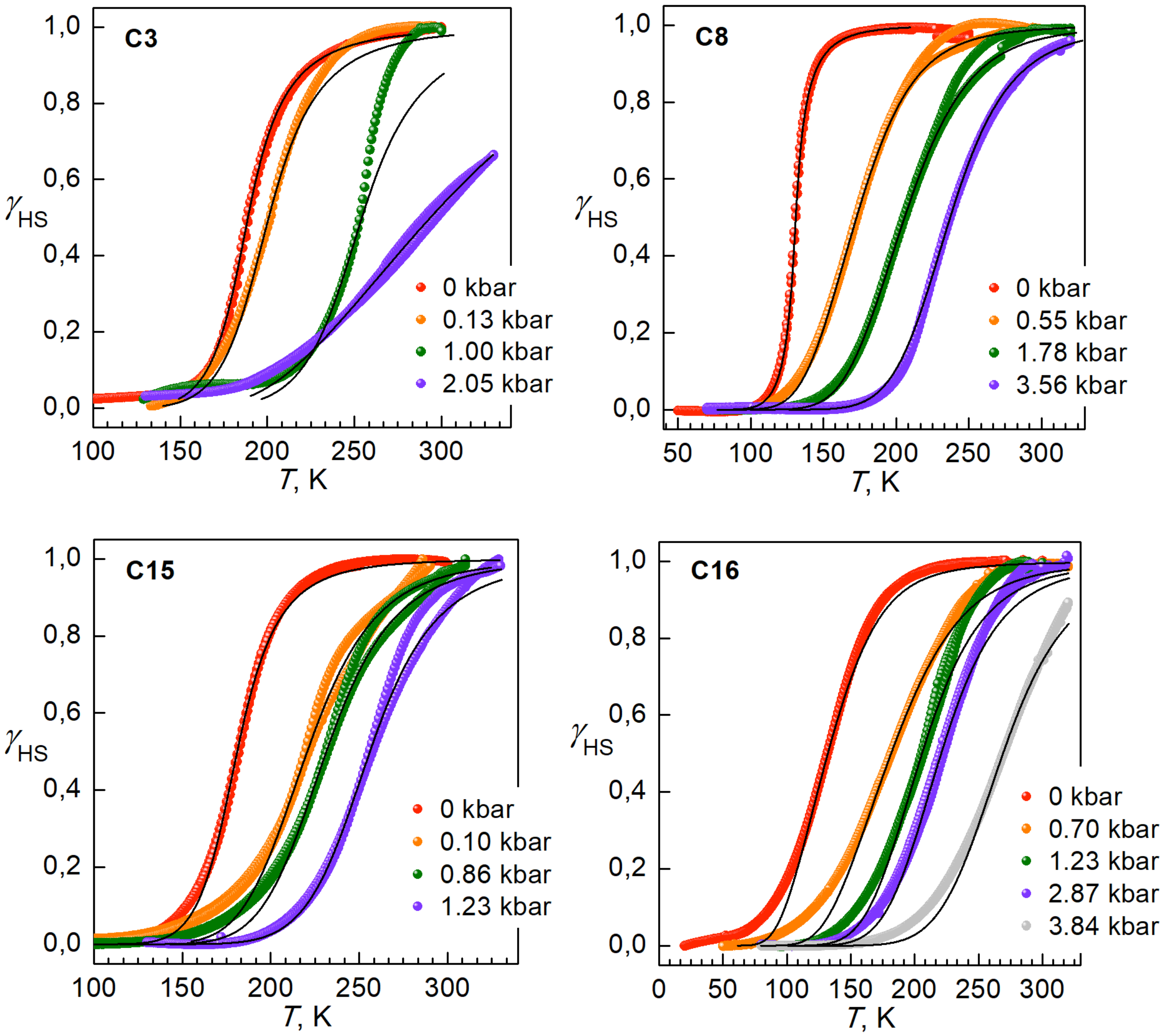

Figure S14. Left: Variation of elastic energy ($\Delta_{elastic}$) and interaction parameter (Γ) with pressure, derived from simulation. Right: Difference between $\Delta_{elastic}$ and Γ, along with the value of ($\Delta_{elastic} - \Gamma$) + $P\Delta V$, under varying pressures for indicated compounds.

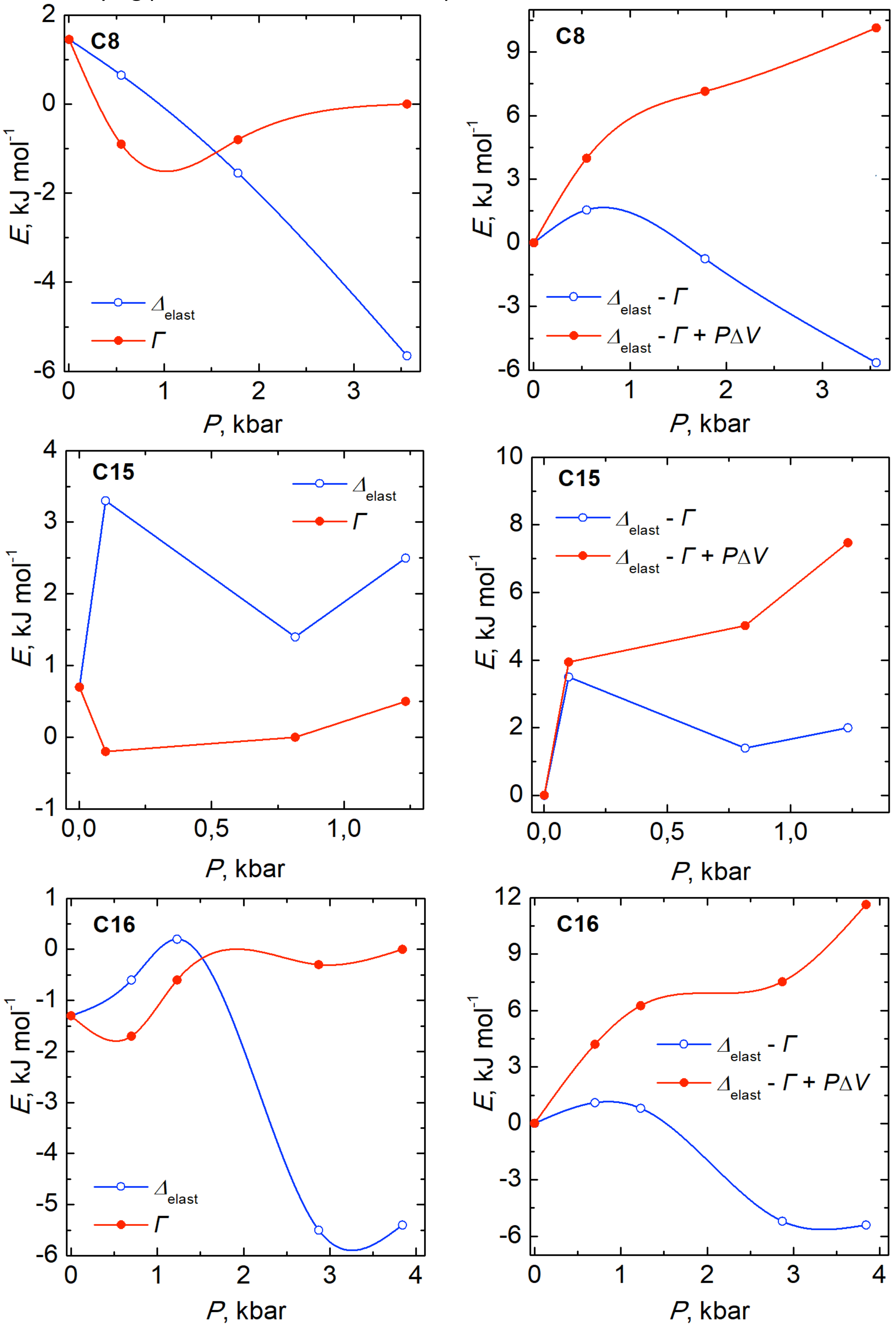

Supplement: Supplementary file 1 [file ic5c03403_si_001.pdf]
